# Statistical analysis of fluorescence intensity transients with Bayesian methods

**DOI:** 10.1126/sciadv.ads4609

**Published:** 2025-04-18

**Authors:** Hamed Karimi, Martin Laasmaa, Margus Pihlak, Marko Vendelin

**Affiliations:** ^1^ Laboratory of Systems Biology, Department of Cybernetics, Tallinn University of Technology, Tallinn, Estonia.; ^2^ Division of Mathematics, Department of Cybernetics, Tallinn University of Technology, Tallinn, Estonia.

## Abstract

Molecular movement and interactions at the single-molecule level, particularly in live cells, are often studied using fluorescence correlation spectroscopy (FCS). While powerful, FCS has notable drawbacks: It requires high laser intensities and long acquisition times, increasing phototoxicity, and often relies on problematic statistical assumptions in data fitting. We introduce fluorescence intensity trace statistical analysis (FITSA), a Bayesian method that directly analyzes fluorescence intensity traces. FITSA offers faster, more stable convergence than previous approaches and provides robust parameter estimation from far shorter measurements than conventional FCS. Our results demonstrate that FITSA achieves comparable precision to FCS while requiring substantially fewer photons. This advantage becomes even more pronounced when accounting for statistical dependencies in FCS analysis, which are often overlooked but necessary for accurate error estimation. By reducing laser exposure, FITSA minimizes phototoxicity effects, representing a major advancement in the quantitative analysis of molecular processes across fields.

## Teaser

Advanced statistical techniques reduce laser damage and improve efficiency in single-molecule fluorescence analysis.

## INTRODUCTION

Fluorescence correlation spectroscopy (FCS) is an established technique used in many disciplines to study the movement of molecules and changes at the single-molecule level (*1*, *2*). The method is based on recording fluorescence intensity transient changes in a small volume using confocal microscopy and analyzing these transients through autocorrelations. Depending on the physical processes causing the fluorescence intensity fluctuations, it is possible to determine diffusion coefficients (DCs) of fluorescent molecules and their concentrations and characterize reactions leading to changes in their fluorescence state (*1*, *2*). As measurements can be performed in live cells, FCS is one of the few methods capable of revealing details about intracellular environment properties under conditions approximating in vivo, thereby providing crucial information for a mechanistic understanding of intracellular processes and their regulation. FCS has been extended by a family of methods to address some of its shortcomings or to study the aspects that were not possible with the classical FCS (*3*–*10*). As the fields progress toward an understanding that the intracellular environment is highly compartmentalized rather than a well-mixed solution (*11*–*13*), resolution requirements for methods probing this environment have increased. However, FCS and related methods use relatively high laser intensities in measurements, which means that performing measurements to explore regional differences in the intracellular environment would require even longer overall exposure of cells to laser radiation, potentially increasing phototoxicity effects (*14*). Thus, alternative methods are needed to study the intracellular environment with reduced exposure to laser illumination.

In addition to high laser illumination requirements, FCS has a well-known flaw in how it is commonly used to obtain properties of the environment or fluorescent molecules. After FCS measurements, the autocorrelation function (ACF) is typically fitted with a model using the least-squares or maximum likelihood methods. This fit is performed under the assumption that the residuals for different lag times are statistically independent. However, this assumption is incorrect and leads to overconfident estimates of model parameters (*15*–*17*). In turn, this has a major implication for using FCS when this assumption is made—it becomes impossible to judge the goodness of fit in a statistically sound manner or compare models of different complexities. For example, it becomes challenging to determine whether the measurements indicate single- or multiple-component diffusion.

One approach to address issues with fitting ACF by FCS models is to account for cross-correlation between residuals corresponding to different lag times. This requires covariance estimation between different lag times, which can be achieved through a larger number of measurements or by using approximation techniques (*15*, *16*). Alternatively, measurements can be repeated many times, composing an overall ACF by taking values for each lag time from a different trace, leading to their independence (*18*). However, while improving the statistical properties of the fits, these methods would require even longer laser exposure than classical FCS, which is often impractical for many experiments.

Several years ago, an alternative framework was proposed by Pressé and colleagues (*19*–*22*). Instead of fitting the calculated ACF of a measurement to a model, they used the Bayesian paradigm and applied the same physical principles as FCS to describe signal formation. In this approach, a Bayesian model was constructed to represent the movement of particles through the confocal volume and fitted against the recorded fluorescence intensity transient. As a result of the fit, posterior probability distributions were obtained for parameters such as diffusion coefficient and molecular brightness. Several variants of the models were tested, all resulting in a remarkable reduction of the required experimental time (*20*, *21*). However, when testing the available implementation of the method (*20*), we found it to be very slow to be used in practice and, as shown in the results of this work, sometimes either failing to converge or converging to an incorrect solution. Thus, while the approach is very promising, major obstacles must be overcome before it can be used in practice.

In this work, we introduce a fluorescence intensity trace statistical analysis (FITSA) method based on the Bayesian paradigm. The implemented method is compared with the model of Jazani *et al.* (*20*), and we demonstrate that FITSA achieves faster and more stable convergence. In addition, we quantitatively compare FITSA and FCS requirements, demonstrating that the same precision of diffusion coefficient estimates can be achieved by FITSA using much shorter experiments than those required by FCS.

## RESULTS

### Overall description of the method

**Fig. 1.**
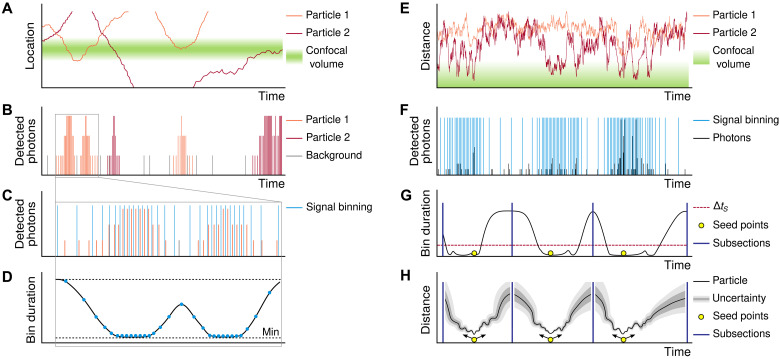
Overall description of the method. Schematic illustration of the FITSA approach for analyzing particle movement near the confocal recording volume. Note that left panels (**A** to **D**) and right panels (**E** to **H**) show different examples to illustrate distinct aspects of the method. (A) Representation of the laser-illuminated confocal volume and movement of particles next to it. (B) Time-resolved photon detection trace recorded by the microscope detection system. (C) Zoomed section of the signal from (B), showing binning based on recorded photon emission rates. (D) Demonstration of smooth transitions between bin sizes, illustrating the adaptive binning approach. (E and F) Normalized distance of particles from the focal point (E) and representative fluorescence intensity trace (F) showing multiple particle transit events through the confocal volume. (G) Segmentation of the signal trace from (F) into subsections, each containing a distinct high-photon detection period. (H) FITSA trajectory reconstruction within a subsection, demonstrating position estimates and their associated uncertainties. Detailed explanation is provided in the main text.

To provide context for the subsequent simulation and experimental results, we present a concise overview of the FITSA method, outlining its underlying principles. In the experiment, a fluorescent particle passes through the laser-illuminated area (Fig. 1A) and generates photons detected by the microscope hardware (Fig. 1B). The detected signal can originate from particles passing through the confocal volume or from background emissions. The FITSA method distinguishes between these signal components by identifying portions of the signal with high photon counts, which contain the most information about particle movement. Signal segments with photon rates higher than the expected background emission rate are isolated for further analysis. This separation enables two distinct analytical approaches: studying overall emitted photons by binning the signal into larger bins to examine background emissions or analyzing rapid changes by tracking short-timescale variations to decode molecular movements.

This dual analytical approach is implemented through the signal binning process. As illustrated in the zoomed area in Fig. 1C, binning tracks the number of photons in each interval. Regions with high photon counts are binned into smaller intervals, while areas with predominantly background emissions are binned into larger intervals. The algorithm creates smooth transitions between these different bin sizes, allowing FITSA to track not only particles within the confocal volume but also their entry and exit (Fig. 1D).

Given the single confocal volume recording, we cannot uniquely determine a particle’s location, as identical photon signals could result from multiple positions within the point spread function (PSF). Instead, we can determine what effectively amounts to a normalized distance from the focal point (Fig. 1E). Under an analytical three-dimensional (3D) Gaussian PSF, this normalized distance varies between lateral and axial directions because of PSF shape asymmetry.

Particle events near the PSF are typically brief and characterized by elevated photon detection rates. Figure 1F schematically shows three possible particle passes through the PSF—these could represent different particles or the same particle entering and exiting during the high-photon detection period. Because particle diffusion is assumed to be independent, FITSA treats these local events as statistically independent and analyzes them separately. This fundamental assumption allows the experimental signal to be divided into distinct subsections for detailed analysis.

Subsection borders are determined by analyzing signal bin durations (Fig. 1G). Regions dominated by background photon emissions, characterized by longer signal bins, serve as natural division points. To estimate particle trajectory and diffusion coefficient, the method selects a seed point at the maximum photon emission within each subsection—typically corresponding to the particle’s closest approach to the focal point. The trajectory calculation begins at the particle’s location in the seed point and follows statistical diffusion properties in both forward and backward time directions. The predicted trajectory shows minimal uncertainty during high-signal transients and maximal uncertainty at subsection boundaries (Fig. 1H).

In the following sections, we demonstrate how this method’s systematic binning, subsection splitting, and trajectory prediction enable robust recovery of diffusion coefficients from short recordings. While the method is described for single-particle contributions to subsection signals, it can be extended to account for multiple-particle scenarios.

### Demonstration with simulated data

**Fig. 2.**
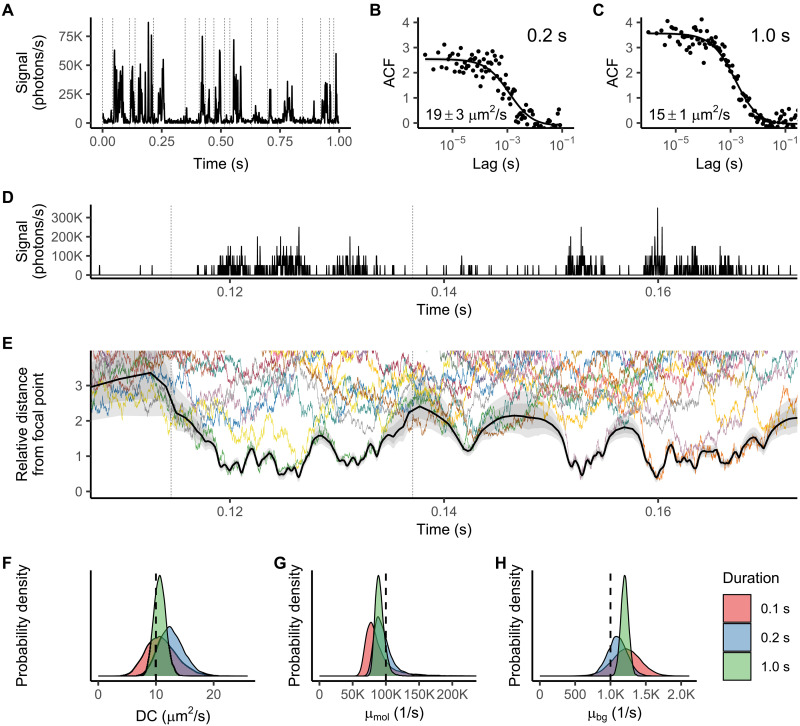
Demonstration of FITSA using the synthetic trace generated with the particles having a diffusion coefficient (DC) of 10 μm^2^/s. (**A**) Fluorescence intensity trace analyzed by FITSA and FCS. In the plot, the signal is reported as an average rate per 1 ms for visibility. Vertical dashed lines denote splitting of the trace into subtraces for FITSA. (**B** and **C**) Time ACFs calculated for the 0.2-s trace start (B) and full trace (C), fitted with the FCS 3D diffusion model. Insets show FCS-derived DCs (mean ± SD). (**D** and **E**) Trace section (D) (average rate per 20 μs) and relative particle distances from the focal point (E). In (E), distances are normalized by PSF waist in each coordinate. Colored lines show particle trajectories used for trace generation; black line (median) with gray areas (±25 and ±40%) shows the FITSA-estimated trajectory. (**F** to **H**) Posterior probability densities for DC (F), molecular brightness (G), and background photon emission rate (H) for different trace durations (0.1, 0.2, and 1 s). Vertical dashed lines indicate true parameter values. Note uncertainty reduction with increased data amount.

To demonstrate the FITSA protocol, we generated a synthetic fluorescence intensity trace (Fig. 2A) by simulating 25 point sources with a diffusion coefficient of 10 μm^2^/s within a small box around the focal point. The trace was relatively short for an FCS analysis, even when correcting for trace duration (*23*). The ACFs calculated for the first 0.2 s and the full 1-s trace were fitted with a 3D motion model, yielding diffusion coefficients higher than the simulation input (Fig. 2, B and C). These ACF fits used FCS models without accounting for the uncertainty heterogeneity or the covariance between ACF values (*15*, *16*), assuming a uniform SD for all ACF values. Consistent with an earlier study (*15*), we found that the classical FCS fit is overfitting the data as it overestimates precision while producing diffusion coefficients that significantly deviate from the true value.

In our FITSA analysis, we examined the synthetic trace (Fig. 2A) in its full length and in shorter segments of 0.1 and 0.2 s. The initial step of FITSA involves dividing the trace into smaller subsections, assuming that a limited number of particles contribute to the signal within each subsection. For this study, we restricted this number to one particle per subsection. Figure 2D illustrates how this approach translates into the identified trajectory by showing a portion of the trace, while Fig. 2E displays the derived trajectory with its associated uncertainty for FITSA performed on the full 1-s trace.

To compare the estimated trajectory with those of all particles used in trace generation, we calculated the relative distance of each particle from the focal point. This distance was found by normalizing contributions of displacements in *x*, *y*, and *z* from the focal point by the corresponding PSF waist dimensions in the same coordinates. After this normalization, it becomes evident that the particle trajectory identified by FITSA closely tracks the particle nearest to the focus at any given moment (Fig. 2E). As demonstrated in Fig. 2E, FITSA switches between tracked particles when one leaves the confocal volume and another enters it (see, for example, the behavior just below 0.16 s in Fig. 2E). In addition, in regions with minimal fluorescence signal, the predicted trajectory exhibits larger uncertainty compared to regions with high signal rates.

This implementation of FITSA generates trajectories along with estimates of the diffusion coefficient, molecular brightness, and background photon emission rate. The resulting posterior distributions for these parameters depend on the length of the analyzed fluorescence trace, as illustrated in Fig. 2 (F to H). As expected, increasing the analyzed trace duration improves the precision of the estimates. While all estimated parameters approximated the true values used in the trace synthesis, closer examination reveals slight deviations of the posterior distribution maxima for both the diffusion coefficient and molecular brightness from the values used in generating the synthetic trace. Such deviations are expected when analyzing very short traces, as demonstrated later in our systematic analysis of both synthetic and experimental data. For background emissions, we observe overestimation of the rate, although the underlying reasons for this bias remain unclear.

Regardless of the trace duration, the true value of the diffusion coefficient consistently fell within the posterior distribution estimated by FITSA. This contrasts with the estimates produced by classical FCS. Such performance demonstrates FITSA’s excellent capability to analyze short traces.

### Comparison with an alternative Bayesian fitting approach

FITSA is similar to the Jazani *et al.* method (*20*) in terms of fitting the fluorescence intensity trace directly by models. We compared FITSA’s performance to the reference implementation of the Jazani *et al.* method (*20*) using the same dataset as in Fig. 2.

**Fig. 3.**
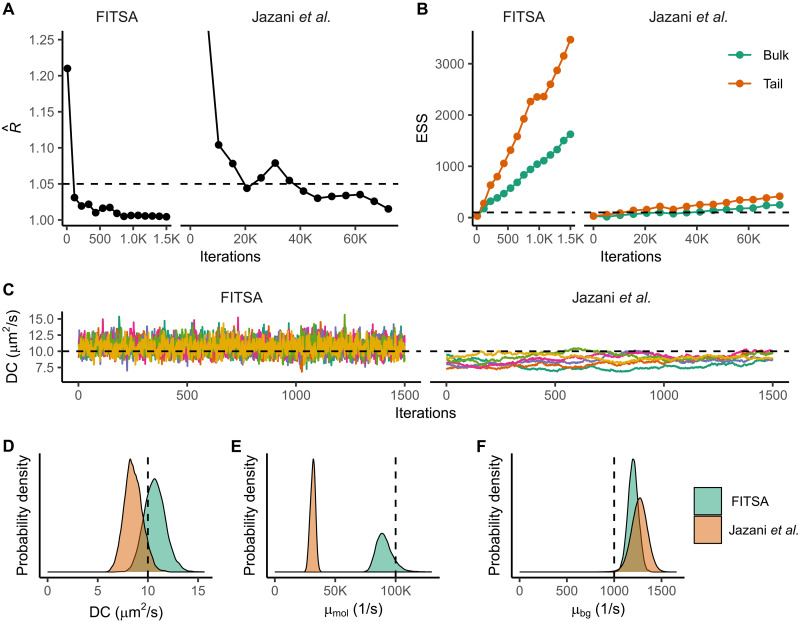
Comparing performance of FITSA with the algorithm by Jazani *et al.* (*20*). Here, the synthetic trace from Fig. 2 was fitted using FITSA or the Jazani *et al.* algorithm. (**A** and **B**) Convergence assessment using potential scale reduction factor $\hat{R}$ (A) and ESSs (B) for iterations after burn-in. Dashed lines show convergence criteria. (**C**) Last 1500 iterations for six chains (color-coded) for FITSA and the Jazani *et al.* method. The dashed line indicates expected DC. (**D** to **F**) Posterior probability densities for DC (D), molecular brightness (E), and background emission rate (F) from FITSA and the Jazani *et al.* method. Vertical dashed lines show true parameter values.

To assess convergence, we performed sampling in six chains, evaluating the potential scale reduction factor $\hat{R}$ and bulk and tail effective sample sizes (ESSs), which quantify the independent information content of the samples and help validate the reliability of posterior estimates (*24*). We used slightly looser convergence criteria than proposed in (*24*), as the Jazani *et al.* method required many iterations to pass even these criteria (Fig. 3, A and B). FITSA needed far fewer iterations than the Jazani *et al.* method. Figure 3C illustrates the last 1500 iterations by six chains for both approaches. FITSA chains show good mixing and sample similar distributions, while Jazani *et al.* samples exhibit strong within-chain correlation, resulting in slow ESS growth and prolonged posterior sampling.

These sampling differences stem from the algorithms used: FITSA uses Hamiltonian Monte Carlo with the No U-Turn Sampler (NUTS), while the Jazani *et al.* method partly uses Gibbs sampling (*20*). Although FITSA’s per-iteration cost is expected to be higher because of NUTS’s calculations, it achieves convergence faster. To compare performance, we determined the number of FITSA iterations required to match Jazani *et al.* solution after 90,000 iterations (18,000 burn-in and 72,000 sampling). FITSA exceeded the convergence parameters ($\hat{R}$ ≤ 1.015, bulk ESS ≥ 250, and tail ESS ≥ 420) after just 600 iterations (300 burn-in and 300 sampling). On the same PC, the Jazani *et al.* method took 101 ± 5 min per chain, while FITSA took 14 ± 2 min (*t* test, *P* < 0.001), making FITSA more than seven times faster. In this comparison, FITSA sampled all chains in parallel, while the Jazani *et al.* implementation was limited to sequential chain sampling. Parallel sampling for the Jazani *et al.* method would have required multiple MATLAB instances, which was infeasible on the test PC because of high random-access memory demands. This sequential testing approach using the Jazani *et al.* implementation inherently disadvantaged the FITSA implementation, as its six chains had to simultaneously share central processing unit cores and random-access memory resources, whereas in the Jazani *et al.* implementation, each chain had exclusive access to PC resources. It is important to note that the performance, as reported here, depends on the underlying algorithms and implementation. For this comparison, we used the reference implementation of the Jazani *et al.* method, as published in (*20*).

Both algorithms produced similar distributions for the diffusion coefficient, encompassing the true value (Fig. 3D). However, the Jazani *et al.* method significantly underestimated molecular brightness, unlike FITSA (Fig. 3E). Both slightly overestimated the background emission rate (Fig. 3F).

We found the Jazani *et al.* algorithm to be sensitive to the molecular brightness prior. For the synthetic trace generated with a diffusion coefficient of 20 μm^2^/s, using priors from (*20*) led to convergence failure (fig. S1). Increasing the prior mean to match the synthetic trace generation value allowed convergence but resulted in underestimated diffusion coefficient and molecular brightness (fig. S2). In contrast, FITSA converged to true values for both priors (figs. S1 and S2) as well as with the priors having mean values of 10,000 and 1000 1/s. FITSA only failed to converge after 1500 burn-in and 1500 sampling iterations when the molecular brightness prior mean was reduced to 100 1/s, a thousand-fold decrease from the true value.

Thus, compared to the Jazani *et al.* method (*20*), FITSA demonstrated superior speed and robustness to prior differences, consistently satisfying strict recommended convergence criteria (*24*) for the studied traces.

### Comparing precision with FCS

To compare FITSA and FCS performance, we used identical data for both methods. We generated synthetic traces with known diffusion coefficients and fitted the same trace segments using either FITSA or FCS. Classical FCS does not account for covariance between ACF values, which can lead to overfitting (*15*). Therefore, we performed two types of FCS estimates: one considering ACF covariance and one without. To determine ACF value covariance, we generated 2000 synthetic traces and calculated ACFs for each trace at time points matching those in the selected segment. From these, we estimated the covariance matrix, which was then used alongside the ACF calculated from the first trace for FCS fitting. To ensure a fair comparison, we used a Bayesian approach for both FITSA and FCS model fits (see Materials and Methods), allowing us to obtain posterior distributions for diffusion coefficient estimates in both cases. In addition, unless stated otherwise, for all FCS fits in this study, we applied corrections for trace duration (*23*) to enhance FCS fits for short traces.

**Fig. 4.**
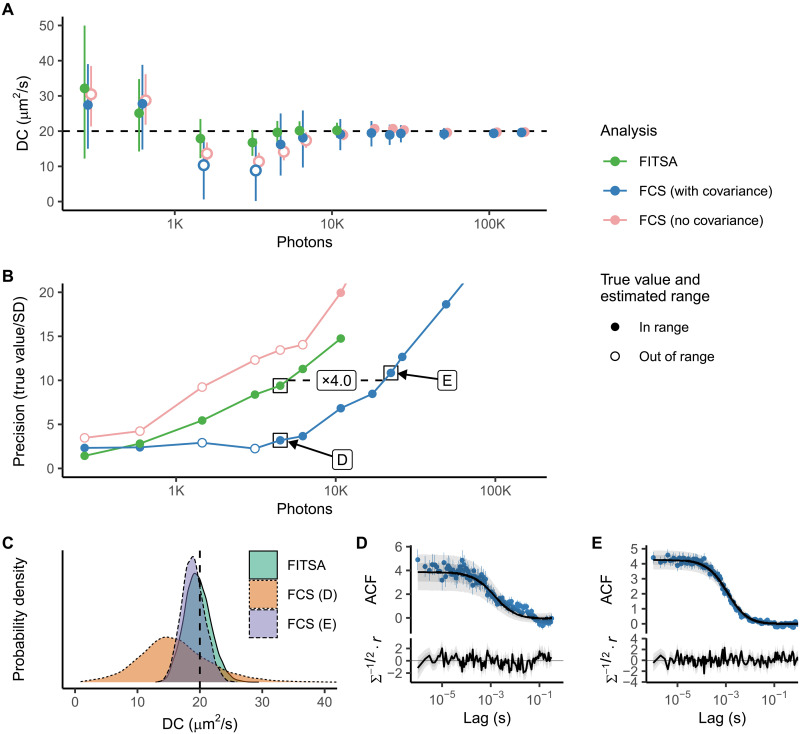
Precision of the estimated DC achieved by FITSA and FCS. Synthetic fluorescence traces were generated with particles having a DC of 20 μm^2^/s. Analysis was performed on the initial section of these traces with varying durations. (**A**) The estimated DC is plotted as a function of photons in the trace exceeding background emission. DC was estimated using FITSA and FCS, with FCS either accounting for ACF covariance (with covariance) or assuming independent ACF values with uniform SD (no covariance). Ranges represent the highest density interval (HDI) of 88%. Points are filled if the HDI contains the true value and the estimated mean value is within the HDI. DC estimates from the same trace are slightly offset to prevent overlap. FITSA estimates DC with shorter traces and demonstrates higher precision than FCS when ACF covariance is considered. See the main text for a discussion on FCS estimates without covariance. (**B**) The precision of DC estimates by different methods is shown as the true value divided by the SD. The same notation is used as in (A). FITSA achieves a relative precision of 10 with shorter traces compared to FCS (with covariance), requiring 4× fewer photons (indicated by the dashed line and label). Estimates marked by black boxes were selected to illustrate the posterior probability density for DC estimated by FITSA (precision close to 10) and FCS for the same trace duration as FITSA or when a precision of 10 was reached. (**C**) Posterior probability densities for DC estimates marked in (B). (**D** and **E**) ACF and its predictive posterior by the FCS model (the solid line is median; ±25 and ±40% regions indicated by gray areas) are shown for selected FCS estimates. Below, standardized residuals calculated using the ACF predictive posterior are found from residual (*r*) and covariance (Σ).

Figure 4A illustrates the performance of different methods in estimating diffusion coefficients. FITSA shows rapid convergence to the true diffusion coefficient value with high precision, even for short traces. FCS estimates depended on whether ACF value covariance was considered. Assuming independent ACF values with uniform variance resulted in high-confidence FCS model estimates but for shorter traces, these estimates often excluded the true diffusion coefficient value. Given the importance of ACF value covariance, we focused on comparing FITSA with FCS fits that account for covariance. As shown in Fig. 4A, FCS estimates with covariance require longer traces to achieve similar confidence as FITSA.

To quantify the photon efficiency of FITSA compared to FCS, we targeted a relative precision of 10 (SD 10 times smaller than the true value). Figure 4B shows the relative precision obtained by different methods. In line with previous findings on FCS overfitting when ignoring ACF value covariance, the simplified FCS model estimated much higher precision than the complete model accounting for covariance (Fig. 4B). FITSA required about four times fewer photons than the complete FCS model to reach a precision of 10 (Fig. 4B). Figure 4C shows the corresponding diffusion coefficient posterior probability densities for FITSA and FCS estimates near the relative precision of 10, which largely overlap [FCS estimate marked as “FCS (E)”]. For comparison, the posterior distribution for the FCS estimate using the same trace length as FITSA was much wider [“FCS (D)” in Fig. 4C]. This demonstrates FITSA’s superior precision with shorter trace lengths.

We repeated this analysis for synthetic traces with diffusion coefficients of 2 and 200 μm^2^/s (figs. S3 and S4). Results were consistent: FITSA achieved the desired precision using shorter traces than FCS when accounting for ACF value covariance. To reach a relative precision of 10, FITSA required 2 (fig. S3) to 11 (fig. S4) times fewer photons than FCS depending on the trace.

For the 200 μm^2^/s diffusion coefficient trace (fig. S3), the difference between FCS and FITSA in required photons was the smallest. In this case, particles passed the focal point quickly, emitting relatively few photons per event. While the photon count was low, the number of events sufficed for ACF composition and covariance estimation, allowing FCS model fitting to recover the diffusion coefficient.

The 2 μm^2^/s diffusion coefficient trace (fig. S4) showed a more pronounced difference, with FITSA recovering the diffusion coefficient using 11 times fewer photons than FCS. For shorter traces, FCS fits accounting for ACF covariance were poor, resulting in wide posterior distributions for estimated diffusion coefficients. FITSA, however, converged to the correct diffusion coefficient and achieved the required precision using a short trace.

**Fig. 5.**
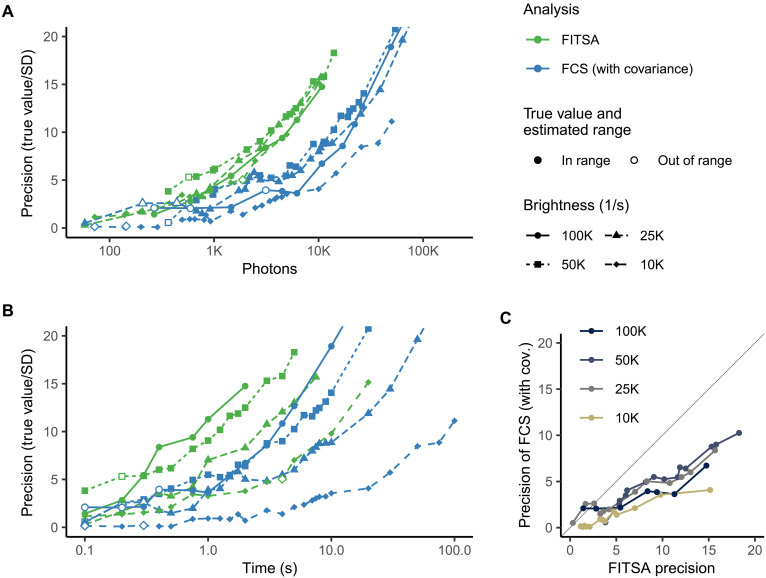
Impact of molecular brightness on estimation precision. As in Fig. 4, synthetic fluorescence traces were generated with particles having molecular brightness reduced from 100,000 1/s to 10,000 1/s and a DC of 20 μm^2^/s. The precision of DC estimation achieved by FITSA and FCS (with covariance) is shown as a function of photons in the trace exceeding background emission (**A**) or the duration of the analyzed trace (**B**). Note that for both methods, precision was mainly determined by the number of photons. In (**C**), the precision of FCS is plotted against the precision of FITSA, following a similar data representation approach as in [(A) and (B)]. Each data point represents a specific simulated condition, and lines connect points obtained under the same molecular brightness, revealing the comparative performance of the two estimation methods across different conditions.

In the previous comparison, the number of photons was considered a primary factor determining the estimation precision. However, the duration of the analyzed signal could also potentially influence the results, as the number of analyzed photons increased with signal duration. To systematically distinguish between the contributions of photon count and signal duration, we conducted an additional analysis by varying molecular brightness from 100,000 1/s to 10,000 1/s (Fig. 5). The comparison of Fig. 5 (A and B) reveals that the number of photons more accurately describes the estimation precision for both FITSA and FCS methods. As illustrated in Fig. 5B, both analytical approaches required progressively longer signal durations when molecular brightness decreased to achieve comparable precision. Notably, across all molecular brightness levels, FITSA consistently demonstrated superior precision compared to FCS, as evidenced in Fig. 5C.

In the comparison above, we demonstrated that FITSA achieves desired precision using several times fewer photons than FCS, assuming known ACF covariance without additional measurements. As discussed later, this improvement likely underestimates FITSA’s actual advantage by orders of magnitude.

### Demonstration with experimental data

**Fig. 6.**
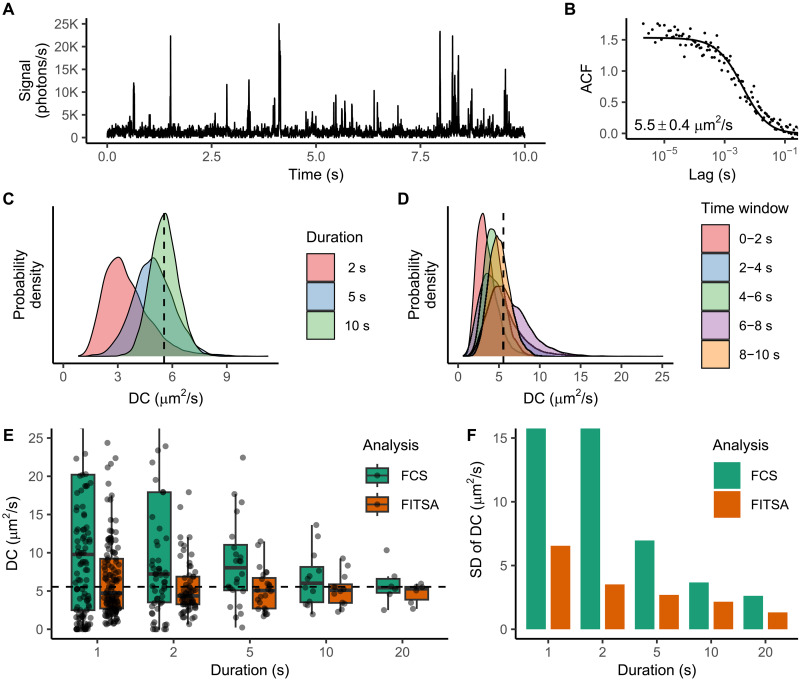
Analysis of the experimental trace recorded for Alexa Fluor 647 Dextran 10K in the 60% glycerol/water mixture. (**A**) Signal obtained during 10 s of 120-s recording for the dye in a 0.1 nM concentration. (**B**) ACF calculated for the 120-s signal trace and fitted by the 3D diffusion FCS model with the estimated diffusion coefficient shown in the inset. In (B), FCS analysis was performed without correcting for the analyzed segment duration, as the experiment’s duration was sufficiently long. (**C**) Diffusion coefficient estimation by FITSA using the first 2, 5, or 10 s of the trace in (A). Note how the posterior of the diffusion coefficient is getting more compact with the increase in the amount of analyzed data. (**D**) For shorter time traces, the diffusion coefficient posterior depends on which part of the signal is analyzed. Here, 2-s traces were taken from the 10-s trace (A) at the different time windows, as indicated by color. (**E**) 120-s trace analyzed by FCS and FITSA using smaller subsections of the trace with the results grouped by the duration of the subsections. For FITSA, the mean value of the diffusion coefficient posterior was shown in this comparison. For clarity, shown diffusion coefficients are limited to 25 μm^2^/s. (**F**) SD of the diffusion coefficient estimations shown in (E) for FCS and FITSA with the vertical axis limited to 15 μm^2^/s for clarity. Note that the spread of the estimated values is considerably smaller for FITSA than for FCS. In [(C) to (E)], the diffusion coefficient value estimated by FCS for the 120-s trace is indicated by a dashed line.

We recorded fluorescence traces under various conditions to demonstrate FITSA’s application to experimental data analysis. In Fig. 6, we show traces and analysis results obtained with Alexa Fluor 647 Dextran 10K in a glycerol-water mixture. The fluorescence peaks are heterogeneously distributed throughout the experiment (Fig. 6A). As more data are analyzed, FITSA’s estimation of the diffusion coefficient becomes more precise (Fig. 6C). However, because of signal heterogeneity, equal trace durations do not necessarily yield identical posterior probability densities for the diffusion coefficient, especially for shorter durations (Fig. 6D).

To characterize estimate variability, we divided a 120-s trace into shorter intervals of different durations. We estimated the diffusion coefficient for each interval using both FITSA and FCS (without covariance) and summarized the results in Fig. 6E. FITSA estimates show notably less variability than FCS estimates. To achieve the same precision as FITSA, FCS would require approximately five times longer measurements. This is illustrated by SD of the estimates for FITSA and FCS at different interval durations (Fig. 6F). Notice that FITSA estimates using 1-s intervals had an SD (6.6 μm^2^/s) comparable to FCS estimates using 5-s intervals (7.0 μm^2^/s), with this trend consistently observed when comparing FITSA estimates at 2 s with FCS at 10 s and FITSA at 5 s with FCS at 20 s.

**Fig. 7.**
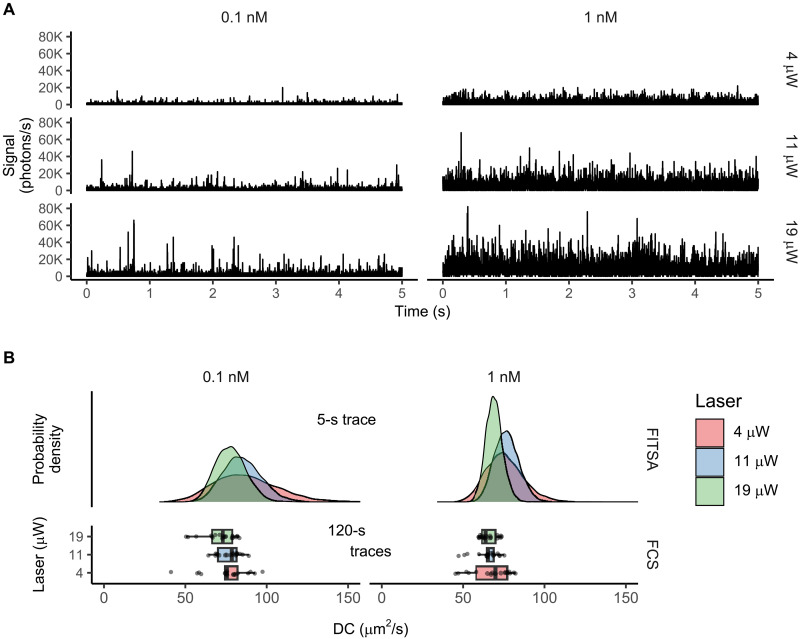
Determination of the diffusion coefficient at different laser powers and Alexa Fluor 647 Dextran 10K concentrations in water. (**A**) Sample 5-s traces analyzed by FITSA and measured with 0.1 nM (left) or 1 nM (right) solutions of Alexa Fluor 647 Dextran 10K at different laser powers (4 to 19 μW from top to bottom). (**B**) Diffusion coefficient posterior probability densities from FITSA (5-s traces, top) compared to FCS results (120-s traces, bottom). For FCS, each point represents one 120-s trace analysis (17 to 18 traces per condition). Here, FCS analysis was performed without correcting for the analyzed segment duration, as the experiment’s duration was sufficiently long. Left and right columns show different dye concentrations. Colors encode laser power. Note the increased estimate precision with higher laser power and concentration.

In a second set of experiments (Fig. 7), we recorded 120-s fluorescence traces with Alexa Fluor 647 Dextran 10K in water, resulting in faster diffusion (~70 μm^2^/s), in agreement with the earlier measurement of 62 μm^2^/s by raster image correlation spectroscopy (*8*) and reported values of diffusion coefficient (60 to 70 μm^2^/s) for similar-sized dextran conjugated with other fluorophores (*25*–*27*). We varied laser power and dye concentration, which strongly affected trace intensity (Fig. 7A). Despite these differences, FITSA-estimated diffusion coefficient posteriors showed similar median values across conditions (Fig. 7B). However, these conditions affected estimate precision for both 5-s traces analyzed by FITSA and 120-s traces analyzed by FCS. Increased laser power led to higher precision in FITSA estimates.

For low dye concentrations (0.1 nM), we carefully selected representative traces to reflect average FITSA estimates. We chose 120-s traces on the basis of FCS-estimated diffusion coefficients close to the median among all estimates for the same concentration and laser power. We then partitioned these traces into 5-s intervals for FITSA analysis, selecting intervals with median FITSA-estimated diffusion coefficients to show as representative posteriors in Fig. 7.

Higher dye concentrations improved precision for estimates from the same time interval because of more particle passage events. However, at a 1 nM concentration, FITSA faced challenges. The current implementation assumes single-particle passages through the confocal volume, which does not hold at higher concentrations. This led to elevated background emission rate estimates. Furthermore, we had to limit the length of subsections used in the fits, as there were no clear phases in the transients corresponding to stages where no particles were near the confocal volume. Consequently, without this imposed limit, the automatic partitioning of the trace was unable to isolate single-particle passages and instead joined multiple passages into a single subsection. This automatic partitioning had been effective in other tests with lower concentrations. Addressing these challenges for higher concentration traces will be the focus of future studies.

**Fig. 8.**
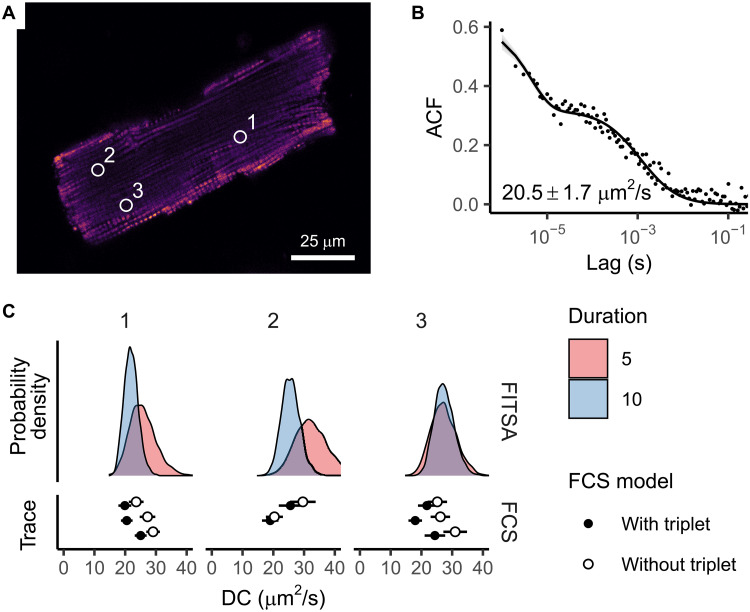
Determination of diffusion coefficients of Alexa Fluor 647 Dextran 10K in the rat cardiomyocyte. (**A**) Confocal image of an adult rat cardiomyocyte depicting mitochondria (MitoTracker Green), along with the diffusion measurement locations of Alexa Fluor 647 Dextran 10K indicated by circles. (**B**) ACF with its predictive posterior calculated using an FCS model that accounts for both diffusion and triplet state dynamics (median shown as a solid line, with ±25 and ±40% probability regions in gray). The ACF was calculated using the last 60 s of the final two traces from location 1 to demonstrate the presence of triplet states. (**C**) Diffusion coefficients determined by FITSA (using 5- and 10-s traces) compared with FCS results (using 60-s traces). FCS estimates are presented as the means with SD for each analyzed trace using models either considering or excluding triplet state contributions. At location “2,” one of three traces was excluded from analysis because of fluorescence instability. Here, FCS analysis was performed without correcting for the analyzed segment duration, as the experiment’s duration was sufficiently long.

In additional experiments, we evaluated FITSA’s ability to determine diffusion coefficients in cardiomyocytes. We created holes in an intact cardiomyocyte by puncturing it using a sharp glass pipette, allowing Alexa Fluor 647 Dextran 10K from the external solution to enter the cell. For each selected cellular location (Fig. 8A), we recorded three 120-s fluorescence intensity traces. For FITSA, we analyzed the middle portion of the second trace (out of three) at each location because it displayed stable fluorescence levels, while, for FCS, we used the final 60 s of all stable traces to estimate the diffusion coefficient (Fig. 8). The first trace typically showed an initial decline in overall fluorescence that stabilized before the second half of recording, except at location “2.” This decline could be attributed to bleaching of weak autofluorescence (observed in experiments without dye), bleaching of dye molecules, or other contributing factors.

In cardiomyocytes, FCS revealed a larger contribution of the triplet state compared to water (Fig. 8B). The fractional population of the triplet state in the studied cardiomyocyte (0.50 ± 0.04, *n* = 6) was significantly larger than in water (0.29 ± 0.04, *n* = 26; *P* < 0.0001, likelihood ratio test). The triplet relaxation time showed no significant difference between the cell (4.1 ± 1.2 μs, *n* = 6) and water (6.0 ± 3.8 μs, *n* = 26; *P* = 0.21, likelihood ratio test). Here, we included only transients that resulted in fits with triplet relaxation times between 2 and 15 μs, avoiding cases where triplet contribution improved ACF fit outside the expected range of 4 to 7 μs for the used dye (*8*). In addition, for the analyzed transient recorded in the glycerol-water mixture (Fig. 6), the contribution of triplet states was negligible, with FCS model fits showing a triplet relaxation time of 1 μs. Thus, compared to the other experimental cases in this study, analysis in cardiomyocytes is complicated by substantial contributions of triplet states.

Despite these challenges, FITSA successfully recovered diffusion coefficients from relatively short transients (10 s), with the posterior distribution of diffusion coefficients encompassing the range predicted by FCS (Fig. 8C). Because current FITSA models do not incorporate triplet states, the predicted posterior of diffusion coefficients shows a slight shift toward larger values compared to FCS models that account for triplet state contribution: 21.8 ± 2.8 μm^2^/s (FCS, *n* = 8) versus 24.9 ± 2.7 μm^2^/s (FITSA posterior medians for 10-s traces, *n* = 3); this difference was not significant (*P* = 0.099, likelihood ratio test). This shift toward larger diffusion coefficients in models without triplet state contribution is expected, as demonstrated by larger diffusion coefficients predicted by FCS (26.5 ± 3.5 μm^2^/s, *n* = 8) when fitting ACF at lag times larger than 2 μs (significant difference between FCS models with and without the triplet state, *P* = 0.005, likelihood ratio test). The estimates by both FITSA and FCS are similar, although slightly higher, compared to previous measurements using raster image correlation spectroscopy (*8*) for the same dye in rat cardiomyocytes: 16 ± 2 μm^2^/s in the transversal direction and 19 ± 3 μm^2^/s in the longitudinal direction. This example demonstrates FITSA’s capability to recover diffusion coefficients in live cells using short fluorescence transients, achieving results similar to those obtained by FCS analysis of 60-s traces.

Looking ahead, we anticipate that extending FITSA models to include transitions between molecular states would improve estimate precision. The development of FITSA models incorporating various physical aspects of real-life measurements will be addressed in future studies.

## DISCUSSION

Fluorescence intensity traces from FCS experiments encode rich information about molecular movement around and within the confocal volume. Recent Bayesian approaches have shown promise in directly analyzing these traces compared to traditional FCS analysis using ACFs (*20*, *21*). Compared to earlier Bayesian approach–based methods (*20*), FITSA converges much faster and demonstrates greater robustness. Rigorous analysis shows that FITSA can estimate diffusion coefficients using much shorter fluorescence traces than FCS, overcoming difficulties in statistical analysis of FCS results.

A major advance of FITSA, enabling it to outperform earlier approaches for direct fitting of fluorescence intensity traces (*20*), stems from its treatment of fluorescence traces as collections of smaller sections. Each section represents an experimental phase where independent particles enter the vicinity of the focal point and subsequently leave it. This conceptual approach of handling the experiment as a series of local events begins with adaptive signal binning based on fluorescent particle proximity to the focal point, facilitating the subsequent division of the transient into smaller sections. Furthermore, FITSA’s strategic selection of seed points at maximum photon emission rates enables efficient sampling of posterior distributions around well-constrained positions, reducing the impact of parameter cross-correlations. These advances result in demonstrably improved convergence speed and greater robustness to priors far from the posterior compared to earlier methods, as evidenced in our validation studies (Fig. 3 and figs. S1 and S2). Both improvements make FITSA more suitable for practical applications and enable the analysis of longer transients than was achievable with earlier approaches (*20*).

The advantages of FITSA’s sectioning approach and strategic seed point selection become evident when considering posterior sampling. When reconstructing particle trajectories, the algorithm first draws coordinates for a seed point and then reconstructs the trajectory by drawing possible particle steps based on elapsed time and proposed diffusion coefficient. Because each consecutive particle position depends on the previous one, the coordinates become strongly correlated throughout the trajectory. Methods analyzing whole transients, as in (*20*), face a substantial challenge: Any change in particle position at the beginning of a trajectory affects all subsequent positions. This challenge is further complicated by varying precision in position estimation—high when particles are near the focal point and low when they are distant, where limited information leads to wide posterior distributions. This creates a particularly difficult sampling problem: When analyzing long trajectories, any large changes in particle coordinates before the particle enters the focal region must remain consistent with the subsequent well-confined positions where the particle produces a measurable signal. FITSA overcomes these challenges by treating particle movements in separate sections as independent events and selecting seed points at maximum fluorescence intensity. This approach confines coordinate cross-correlations within individual sections and starts from positions with minimal posterior uncertainty, substantially improving sampling efficiency and method performance.

FITSA’s sectioning approach also mitigates limitations associated with using unbound diffusion processes in the model. While bound diffusion descriptions exist, their implementation is impractical because of infinite series in coordinate posterior calculations. The unbound diffusion model, used in both FITSA and earlier methods (*20*, *21*), inherently allows particles to drift away from the seed point. FITSA minimizes this effect through its short sections and strategic seed point selection. In contrast, methods analyzing complete transients must compensate for this particle dispersion to maintain particles near the focal point, potentially leading to artificial adjustments such as underestimation of diffusion coefficients, as demonstrated in our comparison with the Jazani *et al.* method (fig. S2).

Before comparing FITSA with FCS, it is important to stress that as pointed out in Introduction, FCS is commonly used assuming statistically independent residuals in ACF values when fitted with models. This assumption is incorrect and leads to data overfitting, as shown in earlier studies (*15*, *16*) and in Fig. 4. To address this, one can estimate covariance between different lag times (*15*) or perform separate measurements for each ACF value (*18*). These approaches are expected to yield better error estimates and enable discrimination between models of different complexities, such as single- or multicomponent diffusion.

When applied to FCS, estimation of covariance or composition of ACF requires a much larger dataset than commonly used and stable preparation throughout the longer experiment required to acquire it. For instance, in our covariance matrix estimation to compare FITSA and FCS performance (Figs. 4 and 5 and figs. S3 and S4), we used 2000 traces for each condition. If we had composed an ACF by taking one point from each trace as in (*18*), we would have used 130 traces in our analysis. These requirements were not accounted for in the comparison between FITSA and FCS. When considering these requirements, FITSA can estimate the diffusion coefficient using 300 to 21,000 times fewer photons than FCS depending on the dataset and approach used to estimate FCS errors.

While the statistical properties of ACF used in FCS have been known for some time, FCS and its derived techniques typically ignore cross-correlation between ACF values in practical applications. Current FCS measurements already require stable background conditions, and extending these measurements 100-fold or more is often impossible in practice. This limitation, considering the wide range of problems where FCS has been applied, makes the determination of intracellular molecular properties, such as diffusion coefficients, a major open scientific challenge. FITSA addresses this challenge in a statistically sound manner and, as we demonstrate with experimental traces (Fig. 6), enables the use of even shorter fluorescence transients than commonly used FCS. This makes it possible to determine diffusion coefficients in cases that were previously considered impractical using FCS with statistically correct ACF fitting. While FITSA does require longer simulation times than conventional FCS, we argue that this is a necessary trade-off for solving the problem in a statistically rigorous way.

From the comparison of FITSA and FCS performance using synthetic data, we observed that their performance gap depends on diffusion coefficient, with FITSA’s advantage over FCS becoming more pronounced for slower diffusion. Taking into account the fact that molecular diffusion in cells is frequently relatively slow—even for smaller molecules such as ATP (adenosine 5′-triphosphate) and cAMP (adenosine 3′,5′-monophosphate), which exhibit diffusion coefficients around 30 μm^2^/s (*8*, *28*, *29*)—FITSA is expected to have a major impact on improving studies of the intracellular environment by reducing the time required for measurements. This reduction in measurement time would consequently minimize laser-induced phototoxic effects on cells during experiments.

Given FITSA’s ability to estimate diffusion coefficients from small amounts of data, it was crucial to recognize the inherent heterogeneity in estimated model parameters because of trace variability. This is illustrated in Fig. 6, where transients of the same duration led to different diffusion coefficient estimates for different short time traces. When comparing FCS performance with FITSA (Fig. 4 and figs. S3 and S4), we ensured that exactly the same transient section was analyzed using a Bayesian approach to fit ACF by the FCS model to obtain confidence levels in estimated diffusion coefficients.

Our approach differs from that used in (*20*), where FCS result variability was analyzed by performing fits on different sections of the trace. Such an approach potentially combines FCS analysis uncertainty with trace heterogeneity. By analyzing the same transient section for both methods, we were able to accurately quantify the performance of FITSA and FCS, isolating methodological differences from trace-to-trace variability. This approach allowed us to precisely estimate the differences in photon requirements between the two methods, providing a more rigorous comparison of their relative efficiencies.

FITSA estimates not only diffusion coefficients but also molecular brightness and background emissions, making it comparable to methods that analyze photon count distributions rather than temporal domain measurements like FCS. The photon counting histogram (PCH) analysis and fluorescence-intensity distribution analysis (FIDA) are two such methods that determine molecular brightness and molecular concentrations in samples (*30*, *31*), and these approaches have been demonstrated to be mathematically equivalent (*32*). An extension of FIDA, called fluorescence intensity multiple distributions analysis (FIMDA), analyzes the same fluorescence trace using photon count histograms obtained at different time intervals. This approach combines the strengths of FIDA and PCH with FCS capabilities, enabling the determination of diffusion coefficients (*33*). To our knowledge, the impact of error covariance in fits for FIDA, FIMDA, and PCH has not been analyzed as extensively as it has for FCS (*15*). However, we anticipate that FIMDA likely faces similar challenges. Specifically, because FIMDA reuses the same underlying recording data by rebinning them at different time intervals, these measurements are unlikely to be statistically independent. Consequently, we expect that ignoring error covariance in FIMDA would affect diffusion coefficient estimations in a manner similar to FCS.

FITSA shares certain similarities with single-particle tracking methods but offers distinct advantages. Most notably, FITSA can effectively analyze particles with much higher diffusion coefficients than those typically studied using single-particle tracking, which is usually limited to coefficients below and around 1 μm^2^/s (although some setups have achieved measurements up to 10 to 20 μm^2^/s) (*34*, *35*). Furthermore, when particle tracking is used to study small molecules, careful consideration must be given to potential optical trapping effects that might influence molecular motion (*36*). This concern is largely mitigated in FITSA and FCS, as these methods record fluorescence changes at a fixed spot rather than tracking individual particles.

In our validation tests, FITSA successfully recovered a diffusion coefficient of 500 ± 35 μm^2^/s from synthetic traces generated with a true value of 500 μm^2^/s. For faster-moving molecules (synthetic trace with 750 μm^2^/s), FITSA showed a slight underestimation, yielding 690 ± 50 μm^2^/s. While we have not tested FITSA with even faster-moving molecules, the main challenge in analyzing such rapid diffusion lies in distinguishing particle passages through the confocal volume from background emissions. This limitation can be addressed by lowering the prior background emission rate during signal preprocessing. Although this adjustment increases computational demands by expanding the number of bins requiring analysis, it enables accurate recovery of diffusion coefficients. Thus, FITSA extends the range of molecular mobility studies beyond what is typically achievable with single-particle tracking methods.

FITSA’s performance depends not only on model equations but also on fluorescence transient preprocessing. While preprocessing parameters can be modified from our tested values, they should be chosen on the basis of the studied system and expected particle dynamics. For slow-moving particles, longer time intervals can be used for binning, reducing computational requirements. On the basis of our testing, time intervals should be substantially shorter than the time a particle takes to traverse the microscope’s PSF volume; otherwise, the diffusion coefficient will be underestimated because of missing information about particle dynamics. The expected background emission rate should not be overestimated, as this would lead to consideration of only larger fluorescence transient peaks. Missing smaller peaks would cause FITSA to underestimate the diffusion coefficient, interpreting the data as particles remaining in the focal point vicinity for longer periods on average. When fluorescence peaks are clearly visible, we used a wide range of data points per subsection with automatic split determination. However, with overlapping peaks from multiple particles, smaller subsection sizes become crucial to analyze independent events separately. These adjustments should still capture particle movement while maintaining sufficient length to avoid missing longer-term dynamics.

FITSA represents a major advancement in shifting from traditional FCS data analysis to direct fitting of fluorescence traces, proving highly effective in many practical applications. However, like most emerging approaches, it has limitations that need to be addressed in future work. The primary challenges we have encountered relate to fitting data from experiments requiring multiparticle models, particularly those involving higher concentrations or multicomponent systems. These challenges manifest in two main aspects. First, the local events, as specified by fluorescence transient subsections, are caused by multiple particles simultaneously passing through the detection volume. A potential solution is to use a nonparametric approach, as demonstrated in previous studies (*20*, *21*), to determine both the number of contributing particles and their trajectories. The second aspect concerns signal analysis when multiple particles contribute continuously to the fluorescence signal. In such cases, there are no periods in the transient that are dominated by background emissions, making it difficult to determine the background emission level from the same transient. Consequently, either the background level must be established in a separate experiment or the dye concentration must be limited. As a practical solution at this stage, until FITSA can handle multiparticle cases, we recommend reducing the concentration of fluorescent dye to levels that are more suitable for analysis in the experimental setup.

Similar to the evolution of FCS over decades, we anticipate that FITSA’s underlying models will require further refinement to better reflect observed phenomena. These refinements include accounting for triplet state formation, chemical reactions, deviations of microscope PSF from the used analytical form, and analysis of background emissions. Because FITSA relies on the same physical principles as FCS, these refinements can build upon numerous previously established studies in the field, accelerating FITSA development. As FITSA analyzes fluorescence traces differently from FCS, further optimization of imaging and other experimental conditions is needed. We expect that these optimizations will greatly improve our ability to study intracellular molecular properties in future studies.

Although FITSA is faster and less memory intensive than previous methods, it still demands considerable computational resources. To address this limitation, we can leverage FITSA’s use of state-of-the-art numerical libraries to potentially accelerate simulations on graphics processing units (GPUs). However, in our test cases, GPU utilization did not yield faster simulation times, suggesting that further optimization of the implementation is necessary to better harness GPU capabilities.

In conclusion, FITSA allows estimation of diffusion coefficients and other parameters from short fluorescence intensity traces. The reference implementation provides a set of applications and model libraries for practical analysis and future development. We anticipate that FITSA and similar approaches will become attractive alternatives for studying molecular events in experiments currently performed using FCS and related techniques. By avoiding correlation calculations and their inherent fitting issues, methods like FITSA that rely on statistical fitting of original datasets are more robust and provide uncertainty estimation for fitted parameters. Given the markedly reduced photon requirements to achieve precision comparable to FCS, we expect that direct data fitting methods will enable more detailed descriptions of intracellular environments and molecular interactions than previously possible.

## MATERIALS AND METHODS

### Model overview

The mathematical model used by FITSA assumes that particles diffuse freely in the 3D space and emit fluorescence because of laser illumination in the confocal microscopy setup. As the model is using a Bayesian approach, its objective is to find the posterior distributions for the parameters of interest given priors and likelihood. In this case, the model estimates the posterior distributions for particle trajectories, their diffusion coefficient *D*, molecular brightness μ_mol_, and background emission rate μ_bg_. For that, the likelihood is formulated using the measured fluorescence intensity trace. To improve convergence, FITSA preprocesses the signal by splitting it into smaller subsections to separate parts where a single particle moves through the confocal volume. The overall likelihood is formulated assuming that each subsection reflects an independent measurement with the particles trajectories being uncorrelated between subsections.

### Signal processing

As outlined in Results, FITSA preprocesses the fluorescence signal through adaptive binning followed by subsection splitting.

#### Signal binning

FITSA preprocesses the fluorescence trace using an adaptive binning strategy that distinguishes between background emissions and particle-originated signals near the confocal volume. The complete binning procedure is illustrated in fig. S8. Consider particles traversing the confocal volume (fig. S8A) and their corresponding detected photon signals (fig. S8B).

The binning process begins with calculating the signal’s rolling average using two time windows: twin1 (10 ms) and twin2 (50 ms). Regions where both rolling averages exceed the expected background emission rate by a binning factor *f*_bin_ (set to 2) are designated as candidates for smaller bins. These regions, termed “accepted areas,” are highlighted in green in fig. S8C, with the acceptance rate defined as *f*_bin_ times the background emission rate.

The signal is then analyzed sequentially in time, with bin size selection adapting to different scenarios. Within accepted areas, bin duration is determined by counting photons until a predefined threshold is reached (*I*_thr_) (fig. S8D). In high–emission rate regions, bin duration is set to the predefined minimum (Δ*t*_min_) when the photon rate would otherwise result in bins smaller than Δ*t*_min_ (fig. S8E). When exiting accepted areas, the photon count is reset, and bin duration is set to either the predefined maximum (Δ*t*_max_) or, at most, bin_increase_ (set to 2) times the previous bin duration (fig. S8F). Upon entering accepted areas, photon counting begins anew, with bin duration determined by either the threshold or maximum duration increase (fig. S8G).

Following the forward analysis, the signal undergoes multiple reverse passes to refine bin transitions, particularly where bin duration changes abruptly upon entering accepted areas. The reverse processing continues until consecutive bins maintain the maximum duration ratio bin_increase_ in both time directions, ensuring smooth transitions throughout the signal. The resulting adaptively binned signal *I*_b_(*t*) features gradual transitions between different bin sizes and serves as input for subsequent analysis.

#### Binned signal splitting

The binned signal was divided into subsections on the basis of bin durations, with splits occurring in regions containing the largest bins, which primarily represent background emissions. The splitting process analyzes the signal in the forward direction, evaluating bin sizes within a moving window of $N_{S}^{\text{min}}$ to $N_{S}^{\text{max}}$ bins. Within this window, the bins exceeding a minimum duration threshold Δ*t*_*S*_ are found. If no bins exceed Δ*t*_*S*_, the bin with the longest duration was selected as a split point. When multiple bins had durations exceeding Δ*t*_*S*_, the first such bin was selected as a candidate. To ensure robust splitting, the algorithm verifies the first potential split point by examining the subsequent $N_{S}^{\text{seq}}$ bins (five used in the current implementation) and selecting the furthest bin within these $N_{S}^{\text{seq}}$ bins that exceeds Δ*t*_*S*_. This process repeats from each established split point until the entire signal is segmented into distinct subsections.

Within each subsection *s*, the algorithm identifies a seed time point $t_{s}^{\text{seed}}$ by locating the maximum of the rolling average calculated over a time window Δ*t*_seed_. This seed point typically corresponds to the moment when a particle is the closest to the confocal volume center.

### Model

#### Likelihood

We assumed that the photon counts in the binned fluorescence trace were described by Poisson statistics (*19*, *20*, *37*, *38*). With this assumption, the likelihood of the observed intensity *I*_b_(*t*_*k*_) for the *k*-th bin is determined as

$$I_{b}\left(t_{k}\right)∼\text{Poisson}\left(\Delta t_{k}\mu_{k}\right)$$ (1)

where Δ*t*_*k*_ = *t*_*k*_ − *t*_*k*−1_ is the duration of the *k*-th bin, and μ_*k*_ is the projected photon emission rate during that bin, as detected by the detector.

#### Emission rate

The emission rate μ_*k*_ is the sum of background emissions and emissions by *N* particles

$$\mu_{k}=\mu_{\text{bg}}+\mu_{\text{mol}}\sum_{n=1}^{N}\text{PSF}\left[x_{n}\left(t_{k}\right),y_{n}\left(t_{k}\right),z_{n}\left(t_{k}\right)\right]$$ (2)

where $x_{n}\left(t_{k}\right)$, $y_{n}\left(t_{k}\right)$, and $z_{n}\left(t_{k}\right)$ are the positions of particle *n* in the 3D space relative to the focal point at time moment *t*_*k*_, and the PSF describes the spatial distribution of detected photons from a single point source (*1*). Here, the *z* axis is parallel to the optical axis of the microscope objective. For the confocal setup used in this study, PSF was approximated by a 3D Gaussian ellipsoid (*1*, *39*)

$$\text{PSF}\left(x,y,z\right)=\text{exp}\left(-2\frac{x^{2}+y^{2}}{{\omega_{\mathrm{xy}}}^{2}}-2\frac{z^{2}}{{\omega_{z}}^{2}}\right)$$ (3)

where ω_*xy*_ is the lateral waist of the PSF, and ω_*z*_ is the axial waist along the *z* axis. Note that in this model, molecular brightness is determined not only by the intrinsic brightness of the particle but also by the detection efficiency of the microscope, which includes the optical system and the detector. The same applies to the background emission rate.

#### Diffusion

Diffusion of particles in the 3D space was defined by propagators (*40*) for each particle

$$\begin{array}{c} x_{n}\left(t_{k}\right)∼\text{Normal}\left[x_{n}\left(t_{k-1}\right),2D\Delta t_{k}\right]\\ y_{n}\left(t_{k}\right)∼\text{Normal}\left[y_{n}\left(t_{k-1}\right),2D\Delta t_{k}\right]\\ z_{n}\left(t_{k}\right)∼\text{Normal}\left[z_{n}\left(t_{k-1}\right),2D\Delta t_{k}\right] \end{array}$$ (4)

Because of the symmetry of the propagators, a chance of observing a sequence of coordinates in the forward direction [as in …, *x*_*n*_(*t*_*k*−2_), *x*_*n*_(*t*_*k*−1_), *x*_*n*_(*t*_*k*_), …] is equal to observing the same sequence in the opposite direction [as in …, *x*_*n*_(*t*_*k*_), *x*_*n*_(*t*_*k*−1_), *x*_*n*_(*t*_*k*−2_), …]. 

#### Priors and inference

We used the same type of priors for the diffusion coefficient, molecular brightness, and background emission rate as in (*20*)

$$\begin{array}{c} D∼\text{Inverse gamma}\left(\alpha_{D},\beta_{D}\right)\\ \mu_{\text{bg}}∼\text{Gamma}\left(\alpha_{\text{bg}},\beta_{\text{bg}}\right)\\ \mu_{\text{mol}}∼\text{Gamma}\left(\alpha_{\text{mol}},\beta_{\text{mol}}\right) \end{array}$$ (5)

where α_*D*_ = β_*D*_ = 1, α_bg_ = α_mol_ = 2.25, β_bg_ = α_bg_/μ_bg_, and β_mol_ = α_mol_/μ_mol_. Used probability density functions are defined in table S1.

Diffusion of the particles was considered separately in each subsection, with coordinates defined from the first time moment of the subsection to the last time moment before the first time moment of the next subsection. Within each subsection, a seed time moment $t_{s}^{\text{seed}}$ was selected as described above, and particle coordinate priors for that time moment were

$$\begin{array}{c} x_{n}\left(t_{s}^{\text{seed}}\right)∼B_{\mathrm{xy}}\cdot \text{Normal}\left(0,1\right)+\left[1+\frac{\left(n-1\right)}{N}\right]\cdot \frac{B_{\mathrm{xy}}}{2}\\ y_{n}\left(t_{s}^{\text{seed}}\right)∼B_{\mathrm{xy}}\cdot \text{Normal}\left(0,1\right)+\left[1+\frac{\left(n-1\right)}{N}\right]\cdot \frac{B_{\mathrm{xy}}}{2}\\ z_{n}\left(t_{s}^{\text{seed}}\right)∼B_{z}\cdot \text{Normal}\left(0,1\right)+\left[1+\frac{\left(n-1\right)}{N}\right]\cdot \frac{B_{z}}{2} \end{array}$$ (6)

where *B*_*xy*_ and *B*_*z*_ were used to shift and scale the priors; the particle index *n* was from 1 to *N*. As the used PSF was symmetric (Eq. 3), priors were set to preferentially shift particles into the positive coordinates. Because of the symmetry of the PSF and the assumption that particles do not interact with each other in the model, our selection of priors does not introduce bias. However, if the PSF is asymmetric or if processes depend on the precise location of particles, such as interparticle interactions, these priors would need to be revised.

Priors for other positions within each subsection *s* were determined on the basis of diffusion propagators (Eq. 4) using the diffusion coefficient as a hyperprior (Eq. 5). For particle positions after the seed time moment, the diffusion propagators were applied according to Eq. 4 in the forward direction. Conversely, for particle coordinates before the seed time moment $t_{s}^{\text{seed}}$, the symmetry of the propagators was used

$$\begin{array}{c} x_{n}\left(t_{k-1}\right)∼\text{Normal}\left[x_{n}\left(t_{k}\right),2D\Delta t_{k}\right]\\ y_{n}\left(t_{k-1}\right)∼\text{Normal}\left[y_{n}\left(t_{k}\right),2D\Delta t_{k}\right]\\ z_{n}\left(t_{k-1}\right)∼\text{Normal}\left[z_{n}\left(t_{k}\right),2D\Delta t_{k}\right] \end{array}$$ (7)

Inference was done by sampling priors for diffusion coefficient, molecular brightness, and background emission rate priors first. Next, priors for particle positions were sampled individually for each subsection and merged to reconstruct particle trajectories across the entire binned signal. Last, using these samples, the likelihood was evaluated. Posterior distributions were then derived on the basis of the likelihood and the priors, as described in the implementation below. In the performed simulations, the number of particles *N* was set to one.

#### Implementation

FITSA is implemented as a set of applications and a library in Python. For FITSA simulations presented here, models were implemented and sampled using NumPyro (*41*, *42*). Sampling was performed by NumPyro’s implementation of NUTS, an extension of the Hamilton Monte Carlo.

The sampling was performed using six parallel chains, consisting of two phases: burn-in and sampling. The burn-in phase comprises initial iterations during which the algorithm stabilizes, while the sampling phase includes iterations used to estimate the posterior distribution. The number of burn-in and sampling iterations was selected to ensure convergence, as indicated by a potential scale reduction factor $\hat{R}$ close to 1 (below 1.05), and reliability of the estimates, verified by bulk and tail ESSs exceeding 100, following standard recommendations (*24*).

The convergence was checked for diffusion coefficient *D*, molecular brightness μ_mol_, and background emission rate μ_bg_. Particle positions were not checked for convergence because multiple combinations of coordinates could yield the same photon emission rate, as determined by the used PSF. In the simulations presented, typically 1500 burn-in and 1500 sampling iterations were used, as these generally met the convergence criteria. However, in the instances with very short transients, the number of sampling and burn-in iterations was increased until convergence was confirmed.

Convergence was checked by processing posterior samples using ArviZ (*43*). In addition, Hamilton Monte Carlo divergences (*44*, *45*) were found to be low during sampling of the formulated model, indicating that the sampler was able to explore the posterior distribution effectively.

### Synthetic traces

Synthetic traces were generated by simulating the Brownian motion of *K* particles inside a box with dimensions *B*_*xy*_ × *B*_*xy*_ × *B*_*z*_ (parallel to the focal plane and along the optical axis). At the beginning of the simulations, particles were randomly seeded in the box following a uniform distribution, and the trajectories were calculated using a specified diffusion coefficient *D*. It was ensured that particles could not cross the boundaries and rebounded upon contact. The size of the box was selected to be sufficiently large to avoid interference through long-term cross-correlations of the signal. The interference was tested by estimating the diffusion coefficient using FCS, and the box was increased if the estimated diffusion coefficient was different from the one used in the simulations.

Fluorescence was sampled using Poisson distribution (Eq. 1) with PSF (Eq. 3) focused at the center of the box, incorporating set molecular brightness and background emission rates. Trajectories and fluorescence trace were computed with a time step of 1 μs, consistent with the experiments described below. Simulations were implemented in Python using NumPy.

### Simulation parameters

Used simulation parameters for synthetic trace generation and FITSA are given in tables S2 and S3, respectively. For synthetic traces analyzed in Results, the same background emission rate was used as was applied during trace generation, while experimental data analysis required background estimation. The initial background level was estimated by assuming that approximately half of the detected photons originated from background emissions. This estimate was then refined through visual inspection of signal binning over a short segment of experimental data (typically 2 s). During this inspection, the background level was adjusted to ensure proper detection of signal peaks, characterized by successful binning of visually identifiable fluorescence intensity increases. The background level was considered appropriately set when the algorithm detected all observed peaks, and this calibrated value was then applied to the entire recording.

For synthetic traces used to test the upper limit of the FITSA detection range, the background level was lowered four times to make sure that particle passages through confocal volume would not be mistaken for background emissions. The parameters governing signal splitting into subsections were selected as follows. $N_{S}^{\text{min}}$ was set to 50 sequential bins to prevent formation of excessively short subsections. $N_{S}^{\text{max}}$ was assigned a large value (1000) to accommodate cases where particles remained near the confocal volume for extended periods. A split point was established when bin durations exceeded Δ*t*_*S*_. Given the selected *I*_thr_ value of 1 used for binning, this criterion indicated periods without photon detection. We assumed that if no photons were recorded within the millisecond range (1 ms for slower particles and 0.5 ms for faster particles), then the particle had left the vicinity of the focal point, and any subsequent peaks were attributed to different particles. For one specific case with a reduced molecular brightness of 10000 1/s, we increased $N_{S}^{\text{min}}$ to 150 to prevent FITSA from splitting the signal into excessively short subsections. The maximal number of bins in subsection $N_{S}^{\text{max}}$ was reduced to 200 in the analysis of 1 nM dextran diffusion in water to prevent merging of the segments. This parameter combination provided FITSA with sufficient flexibility to accurately recover diffusion coefficients across the wide range of values investigated in this study.

### Fluorescence correlation spectroscopy

The FCS data were analyzed by fitting the ACF with a model corresponding to 3D free diffusion of a fluorescent particle without triplet states (*46*). Here, ACF *G*(τ) (*2*, *46*) was found from the experimental fluorescence trace

$$G\left(\tau \right)=\frac{\left\langle \delta I\left(t\right)\delta I\left(t+\tau \right)\right\rangle }{{\left\langle I\left(t\right)\right\rangle }^{2}}$$ (8)

where δ denotes fluctuations, and τ is the lag time.

For a single component diffusing in three dimensions, without considering the triplet state, *G*(τ) is

$$G\left(\tau \right)=\frac{1}{V_{\text{eff}}\left\langle C\right\rangle }\frac{1}{1+\frac{4D\tau }{{\omega_{\mathrm{xy}}}^{2}}}\frac{1}{\sqrt{1+\frac{4D\tau }{{\omega_{z}}^{2}}}}$$ (9)

where ⟨*C*⟩ is the average concentration of fluorescent molecules in the focal volume, and *V*_eff_ is the effective focal volume (*1*). The effective volume is defined by PSF (Eq. 3)

$$V_{\text{eff}}=\frac{{\left[\int \text{PSF}\left(r\right)dV\right]}^{2}}{\int {\text{PSF}}^{2}\left(r\right)\mathrm{dV}}$$ (10)

where integration is performed around the focal point (*47*). When analyzed considering the contribution of the triplet state, the compensation factor

$$1+\frac{T}{1-T}\text{exp}\left(-\frac{\tau }{\tau_{T}}\right)$$ (11)

was incorporated into *G*(τ) as in (*1*, *8*). Here, *T* is the fraction of molecules in triplet state and τ_*T*_ is the triplet state relaxation time.

In this work, FCS analysis was applied to relatively short segments. Unless specified otherwise, to prevent bias in estimating the diffusion coefficient, we implemented corrections accounting for shot noise and photon count correlations within the segment (*23*). The second-order binning function used to correct photon counts within the segment for the 3D observation volume was taken from (*48*).

To compare estimated diffusion coefficients between FITSA and FCS, a Bayesian approach was used to fit ACF with the model, as described in (*15*). The fits were conducted either by assuming that ACF consists of independent individual measurements or by using a general multivariate Gaussian distribution with covariance (*15*). Wide, noninformative priors were used for the diffusion coefficient and concentration.

The FCS models were implemented in Python using three different approaches. Least-squares fitting with the Trust Region Reflective algorithm (*49*) was used for the analysis presented in Fig. 6E. For Bayesian analyses, we used either UltraNest (*50*) or PyMC (*51*) implementations. The UltraNest implementation was used for analyses shown in Figs. 2, 6B, 7, and 8, while the PyMC implementation was applied to all other analyses, specifically those considering ACF covariance.

### Experiments

#### Confocal setup

Measurements were performed using a custom confocal setup described in (*8*) using a water-immersion 60× objective (UPLSAPO; numerical aperture, 1.2; Olympus). For diffusion measurements, a 633-nm laser (05-LHP-151, Melles Griot, US) was focused on the sample. Emission was detected through a long-pass filter (F76-631, Semrock, Rochester, NY) by an avalanche photodiode (SPCM-AQRH-54, Excelitas Technologies, Pittsburgh, PA) and recorded by a data acquisition card (PCIe-6353, National Instruments, Austin, TX) every 1 μs. For imaging mitochondria, a 488-nm laser (0488L-11A-NI-NT-NF, Integrated Optics UAB, Lithuania) was used to excite MitoTracker Green–stained cells and emission was collected through a 550/88-nm filter (FF01-550/88-25, Semrock, Rochester, NY) at a sampling rate of 10 μs.

#### Experiments on solutions

Experiments were performed using Alexa Fluor 647 Dextran 10K in water or glycerol/water mix at room temperature (22°C). Used concentrations were 1 and 0.1 nM, diluted in the presence of 0.5‰ Tween-20, as suggested in (*52*).

#### Experiments on cardiomyocytes

All animal procedures were carried out according to the guidelines of Directive 2010/63/EU of the European Parliament on the protection of animals used for scientific purposes and had been approved by the Project Authorisation Committee for Animal Experiments in the Estonian Ministry of Rural Affairs (permission no 1.2-17/169, 2023).

Freshly isolated rat (Wistar Han, female, 257 days old; animal supplier: Envigo RMS, 5961 NM Horst, the Netherlands) ventricular cardiomyocytes used to determine diffusion coefficients in different locations in the cell. Cells were isolated as described in (*53*). Before a measurement, isolated cells were first stained with MitoTracker Green FM (M7514, Invitrogen, Eugene, OR) with a final concentration of 250 nM for 10 min to label mitochondria. Then, stained cells were placed in a reusable silicon insert (94.6077.434, flexiPERM, SARSTEDT AG & Co. KG, Nümbrecht, Germany) attached to a coverslip containing intracellular fluid mimicking solution. The solution contained 0.5 mM EGTA, 3.0 mM KH_2_PO_4_, 3.0 mM MgCl_2_, 20 Hepes, 110 mM sucrose, 20 mM taurine, 0.5 mM dithiothreitol, 60 mM lactobionate, 5 mM glutamate, 2 mM malate, 5.0 mM MgATP, 10 mM PCr, and 10 nM Alexa Fluor 647 Dextran 10K. In addition, bovine serum albumin (5 mg/ml) was added, and pH was adjusted to 7.1 at 25°C with KOH.

After about 5 min of cell sedimentation in the silicon insert under the microscope, a cell attached to the coverslip was located. The outer membrane of the cell was then mechanically permeated using a glass microneedle, which was controlled by a micromanipulator (SMXS-K-L-EUR, Sensapex, Oulu, Finland). The microneedle, with a tip size of around 0.5 μm, was made from 1.0-mm-diameter glass rods (TW100F-3, World Precision Instruments, Sarasota, US) using a pipette puller (PC-10, Narishige, Japan). Following the permeation, the internal and external environments of the cell were allowed to equilibrate for 5 min. First, a confocal image of adult rat cardiomyocyte mitochondria was recorded, after which different locations inside the cell were selected for diffusion measurements.

#### PSF estimation

The microsphere slides for measuring PSFs were prepared as in (*54*). Briefly, a 10,000-fold dilution of the original suspension of microspheres (TetraSpeck, T7279, Invitrogen, Eugene, OR) was made using water. A small drop of this diluted solution was then placed on a cover glass with a thickness of 0.17 mm and allowed to air dry. Once the sample was dry, a small drop of immersion oil (Carl Zeiss Immersol W, Oberkochen, Germany), with a refractive index of 1.334 at 23°C, was added to the spot and secured with a glass slide.

Under the microscope, an area containing several microspheres was selected so that Airy patterns would not overlap on either side of the focus. For measurement, the voxel size in the *xy* plane was set to less than 40 nm and, in the *z* plane, it was 100 nm, and several *z*-stacks were recorded. From the *z*-stacks, point sources were identified and averaged according to center positions found from the least-square fitting with Eq. 3. Last, the average point source was fitted with Eq. 3 to determine the PSF lateral waist and axial waist used in analysis.

### Statistics

If not stated otherwise, statistics are reported using the means ± SD. For experiments repeated on the same location several times, we used linear mixed models to quantify the impact of the fixed factors.The models were composed with random intercepts, location considered as random factor. To determine the significance of the fixed factor(s) and their interaction(s), we composed models with and without the corresponding factor and *P* values were obtained by the likelihood ratio test of the full and simplified models. Statistical analysis was performed in R using lme4 (*55*) for linear mixed model analysis. *P* < 0.05 was considered statistically significant.

### Use of artificial intelligence tools

Text was edited with the assistance of large language models: Claude 3.5 Sonnet (Anthropic, San Francisco, CA) and ChatGPT 4.0 (OpenAI, San Francisco, CA). Artificial intelligence tools were used for correcting grammar and improving the clarity of the text sections. The full prompt used was as follows: “I would like to ask you to help with English and edit text for clarity if needed,” with the corresponding text added after this prompt. Before incorporating any artificial intelligence–suggested changes into the manuscript, all proposed edits were reviewed by the authors to ensure that the original meaning was preserved.
