# Supplementary material for: Statistical analysis of fluorescence intensity transients with Bayesian methods
Source: Sci Adv. 2025 Apr 18;11(16):eads4609. doi: 10.1126/sciadv.ads4609 (PMC12007579; doi:10.1126/sciadv.ads4609)
Supplement: Supplementary file 1 — Figs. S1 to S8 Tables S1 to S3 [file sciadv.ads4609_sm.pdf]

Science Advances
AAAS

Supplementary Materials for

Statistical analysis of fluorescence intensity transients with Bayesian methods

Hamed Karimi *et al.*

Corresponding author: Marko Vendelin, markov@sysbio.ioc.ee

Sci. Adv. **11**, eads4609 (2025)
DOI: 10.1126/sciadv.ads4609

This PDF file includes:

Figs. S1 to S8
Tables S1 to S3

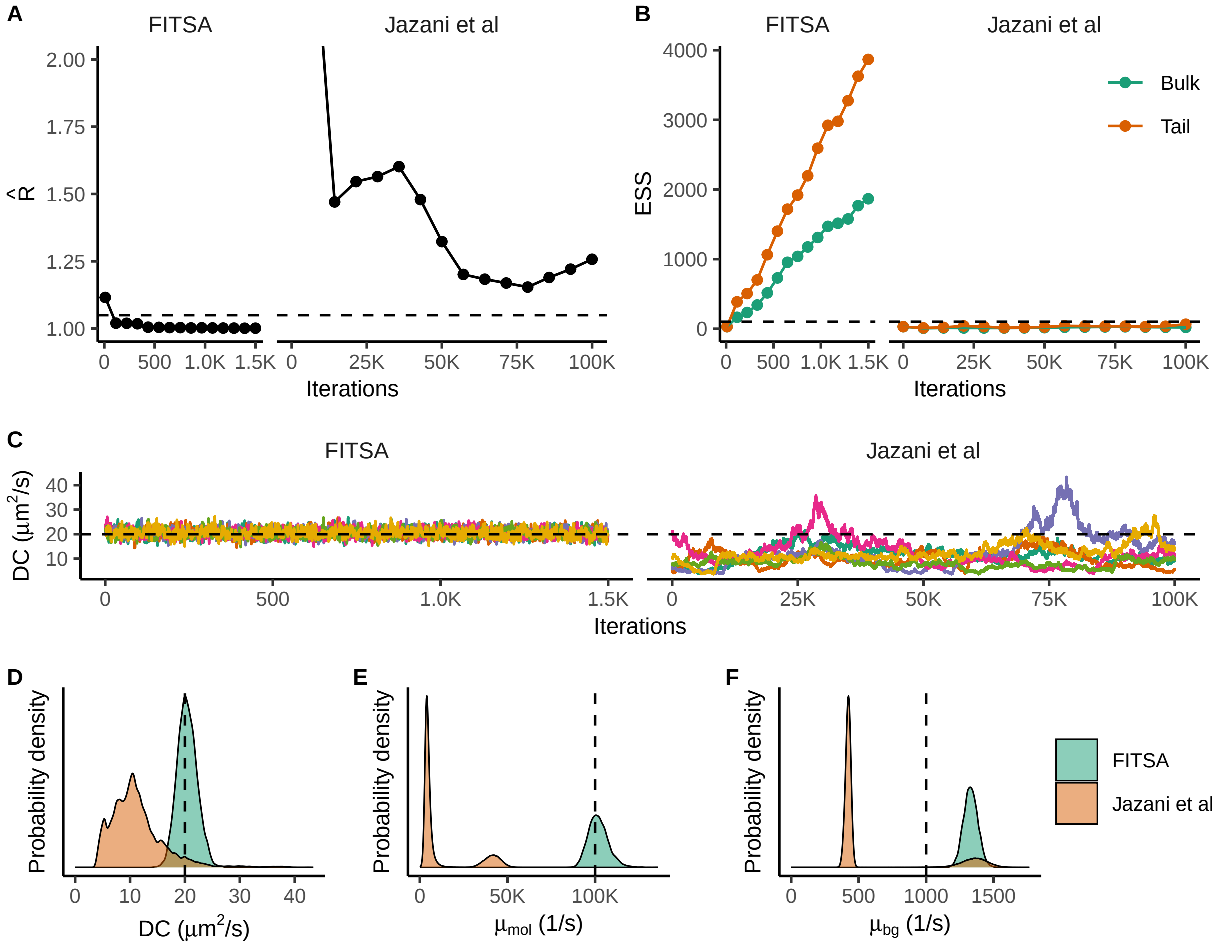

Figure S1: **Comparing sensitivity of FITSA with the algorithm by Jazani et al (20) to the prior with underestimated molecular brightness.** In contrast to Fig 3, synthetic trace was generated with the particles having the diffusion coefficient of $20\,\mu m^2/s$. When fitting, the molecular brightness prior was set with the mean value of $20000\,s^{-1}$. A, B: Convergence assessment using potential scale reduction factor $\hat{R}$ (A) and effective sample sizes ESS (B) for iterations after burn-in. Dashed lines show convergence criteria. C: DC sampled in 6 chains by FITSA and Jazani et al after burn-in iterations. Here, 1500 and 100000 iterations were used as a burn-in for FITSA and Jazani et al, respectively. D, E, F: Posterior probability densities for diffusion coefficient (D), molecular brightness (E), and background emission rate (F) from FITSA and Jazani et al. Vertical dashed lines show true parameter values. Note that sampling by Jazani et al algorithm fails to converge (too large $\hat{R}$ in A, too low ESS in B), in agreement with the differences between sampling in different chains (C).

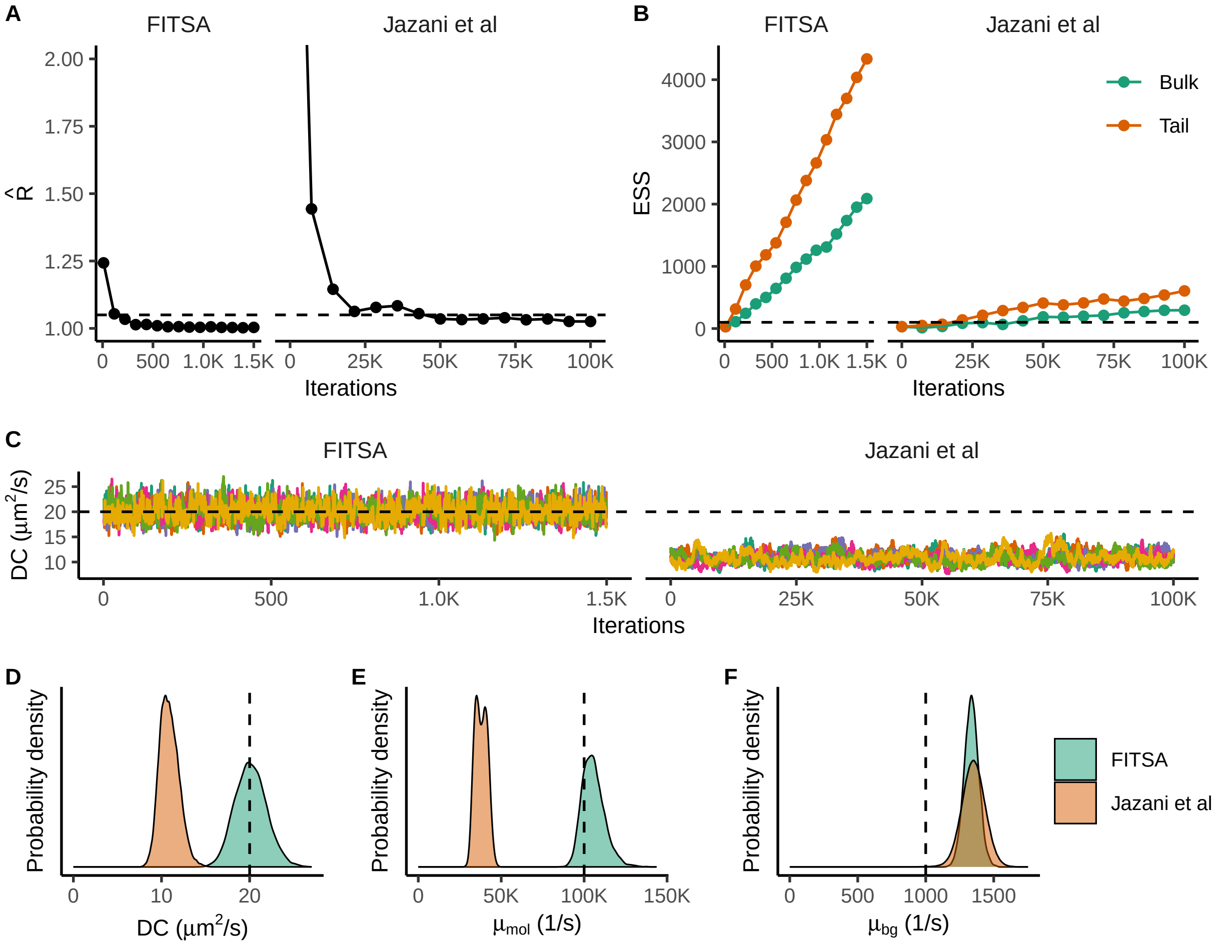

Figure S2: **Comparing performance of FITSA with the algorithm by Jazani et al (4) with the molecular brightness prior having the same mean value as the brightness used to generate the synthetic trace.** As in Fig S1, synthetic trace was generated with the particles having the diffusion coefficient of $20\,\mu m^2/s$. A, B: Convergence assessment using potential scale reduction factor $\hat{R}$ (A) and effective sample sizes ESS (B) for iterations after burn-in. Dashed lines show convergence criteria. C: DC sampled in 6 chains by FITSA and Jazani et al after burn-in iterations. Here, 1500 and 100000 iterations were used as a burn-in for FITSA and Jazani et al, respectively. D, E, F: Posterior probability densities for diffusion coefficient (D), molecular brightness (E), and background emission rate (F) from FITSA and Jazani et al. Vertical dashed lines show true parameter values. Note that while sampling by Jazani et al algorithm converges, it significantly underestimates the diffusion coefficient and the molecular brightness.

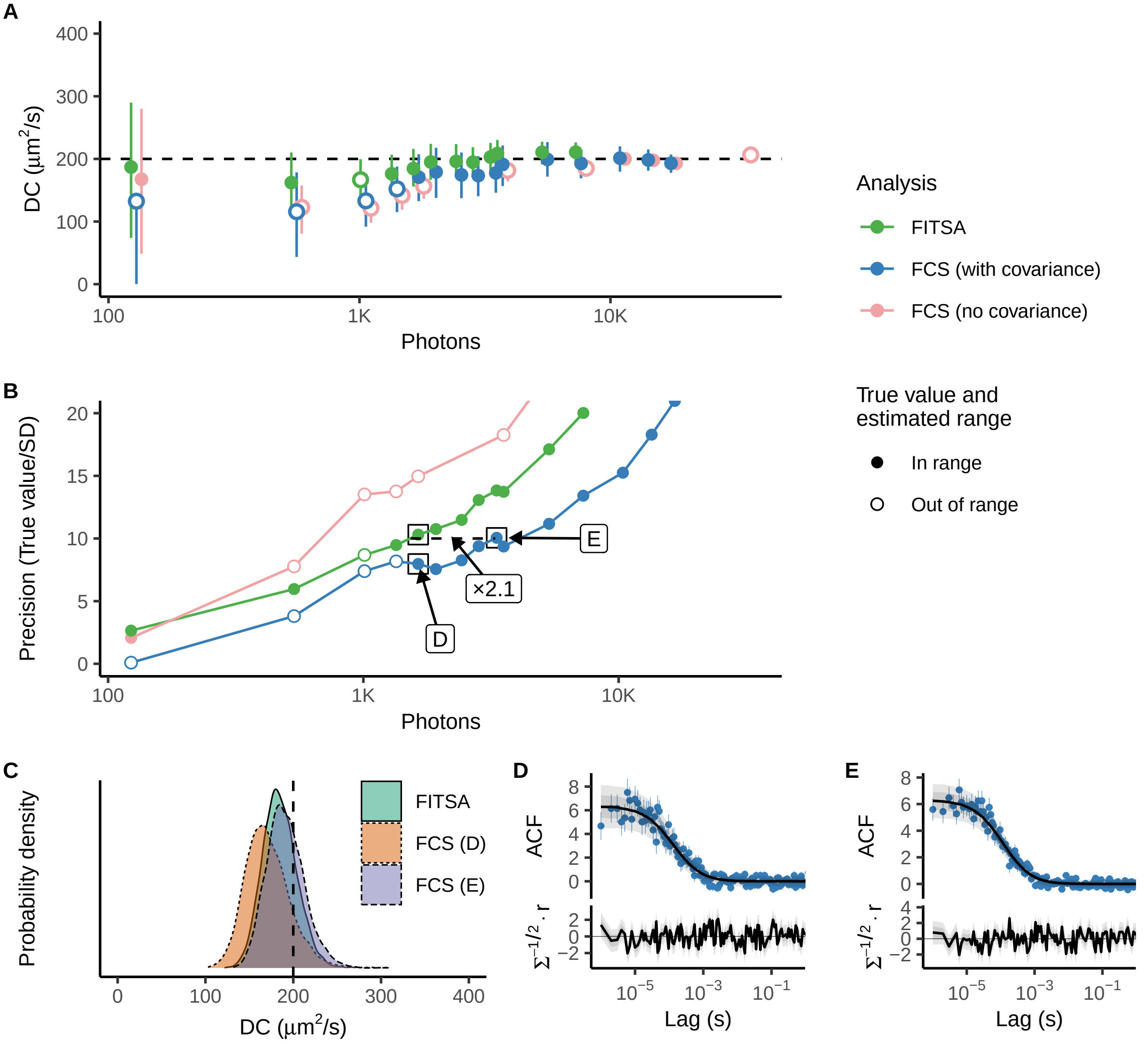

Figure S3: **Precision of estimated diffusion coefficient achieved by FITSA and FCS using synthetic trace corresponding to diffusion coefficient of** $200\ \mu m^2/s$**.** Here, the same notation and analysis presentation as in Fig 4 are used. A: The estimated DC is plotted as a function of photons in the trace exceeding background emission. B: Precision of DC estimates by different methods is shown as the true value divided by the standard deviation (SD). Estimates marked by black boxes were selected to illustrate the posterior probability density for DC estimated by FITSA (precision close to 10) and FCS for the same trace duration as FITSA or when precision 10 was reached. C: Posterior probability densities for DC estimates marked in B. D, E: ACF and its predictive posterior by FCS model are shown for selected FCS estimates. Below the fit, standardized residuals are shown (r – residual; Σ – covariance).

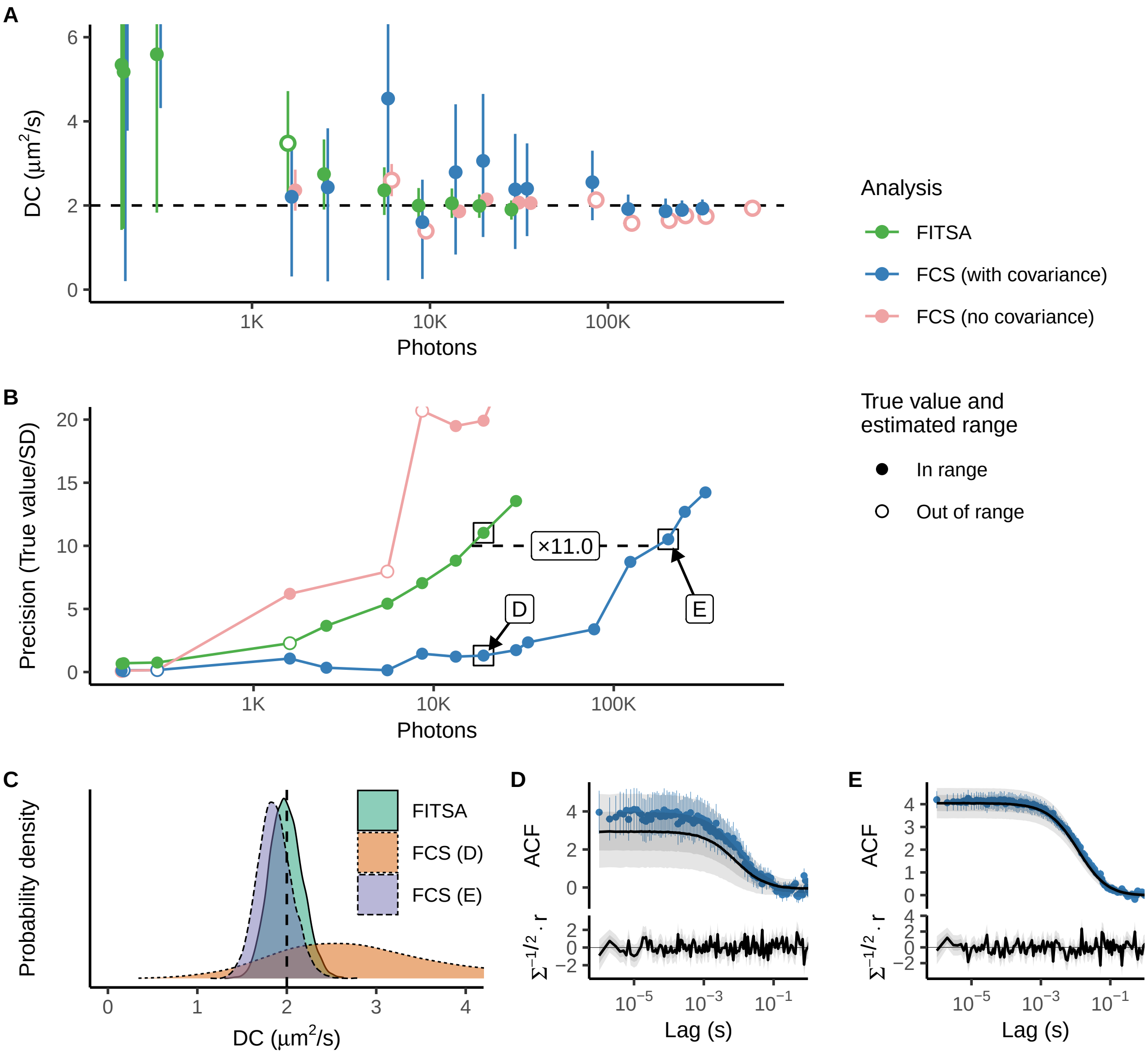

Figure S4: **Precision of estimated diffusion coefficient achieved by FITSA and FCS using synthetic trace corresponding to diffusion coefficient of** $2\,\mu m^2/s$**.** Here, the same notation and analysis presentation as in Fig 4 are used. A: The estimated DC is plotted as a function of photons in the trace exceeding background emission. B: Precision of DC estimates by different methods is shown as the true value divided by the standard deviation (SD). Estimates marked by black boxes were selected to illustrate the posterior probability density for DC estimated by FITSA and FCS. C: Posterior probability densities for DC estimates marked in B. D, E: ACF and its predictive posterior by FCS model are shown for selected FCS estimates. Below the fit, standardized residuals are shown (r – residual; Σ – covariance).

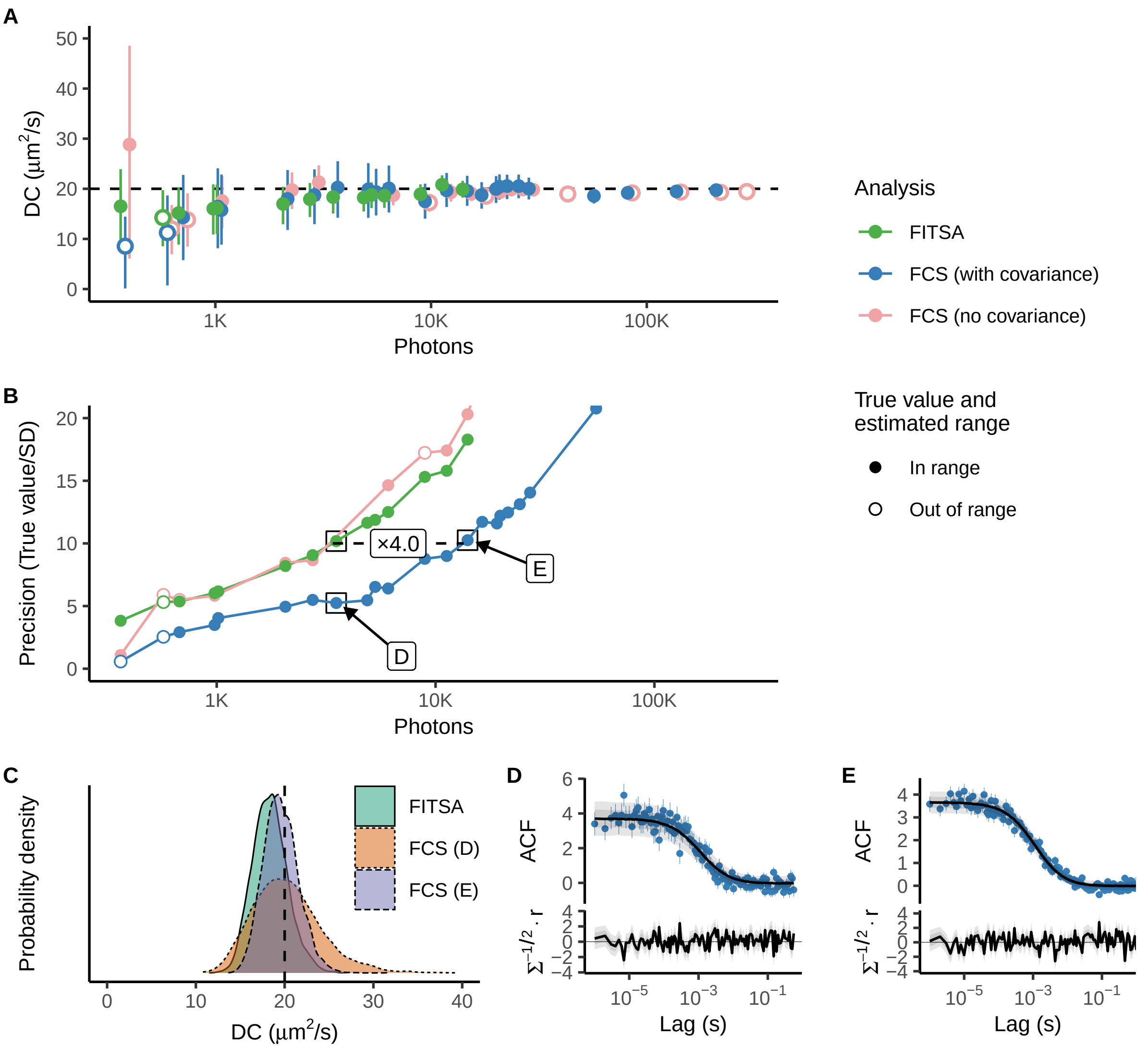

Figure S5: **Precision of estimated diffusion coefficient with the reduced molecular brightness.** Here, a synthetic trace corresponding to diffusion coefficient of 20 $\mu m^2/s$ was used with the molecular brightness of 50000 1/s. These data were used while composing a summary of molecular brightness impact shown in Fig. 5. A: The estimated DC is plotted as a function of photons in the trace exceeding background emission. B: Precision of DC estimates by different methods is shown as the true value divided by the standard deviation (SD). Estimates marked by black boxes were selected to illustrate the posterior probability density for DC estimated by FITSA and FCS. C: Posterior probability densities for DC estimates marked in B. D, E: ACF and its predictive posterior by FCS model are shown for selected FCS estimates. Below the fit, standardized residuals are shown (r – residual; Σ – covariance).

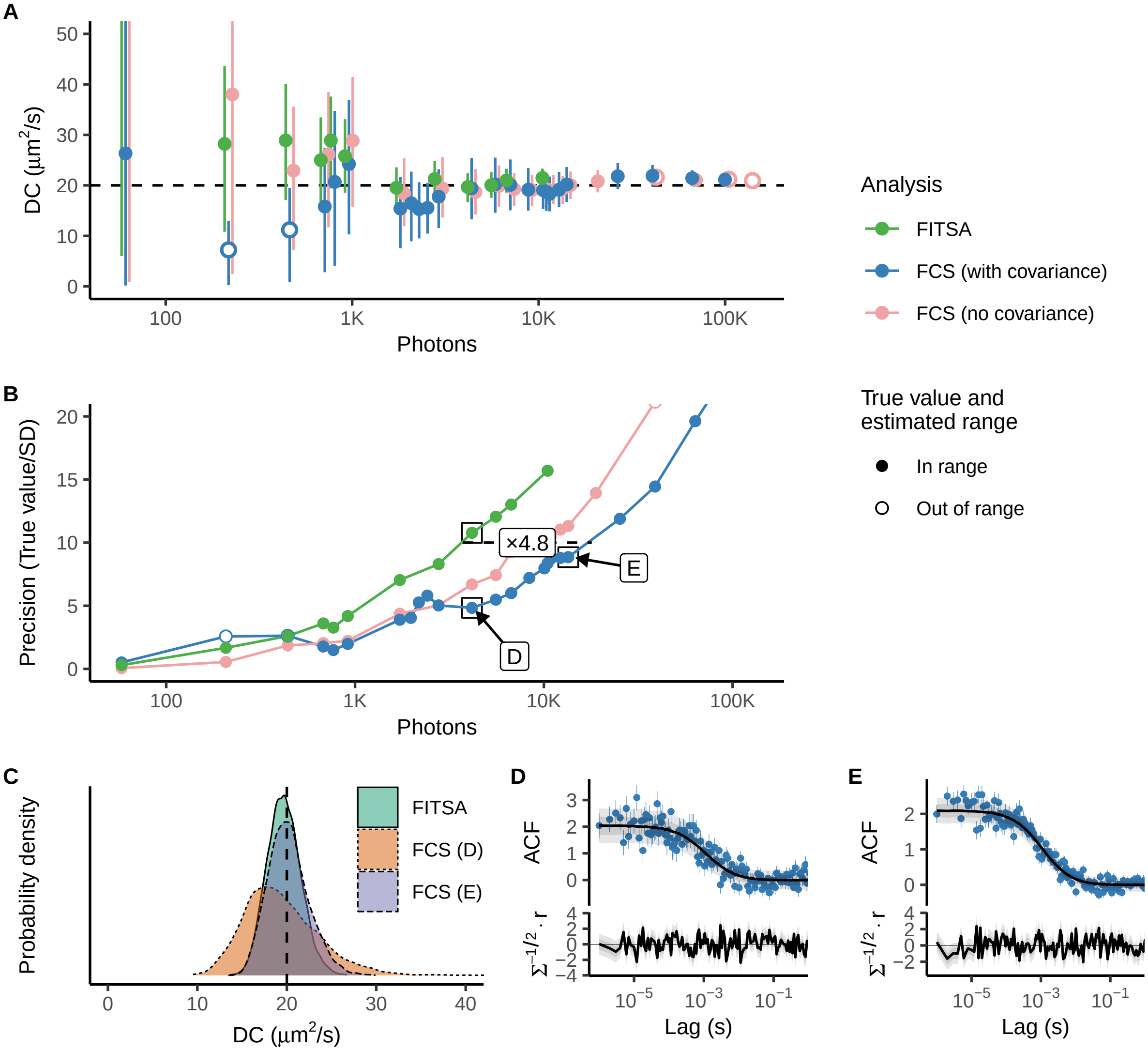

Figure S6: **Precision of estimated diffusion coefficient with the reduced molecular brightness.** Here, a synthetic trace corresponding to diffusion coefficient of $20\,\mu m^2/s$ was used with the molecular brightness of 25000 1/s. These data were used while composing a summary of molecular brightness impact shown in Fig. 5. A: The estimated DC is plotted as a function of photons in the trace exceeding background emission. B: Precision of DC estimates by different methods is shown as the true value divided by the standard deviation (SD). Estimates marked by black boxes were selected to illustrate the posterior probability density for DC estimated by FITSA and FCS. C: Posterior probability densities for DC estimates marked in B. D, E: ACF and its predictive posterior by FCS model are shown for selected FCS estimates. Below the fit, standardized residuals are shown (r – residual; Σ – covariance).

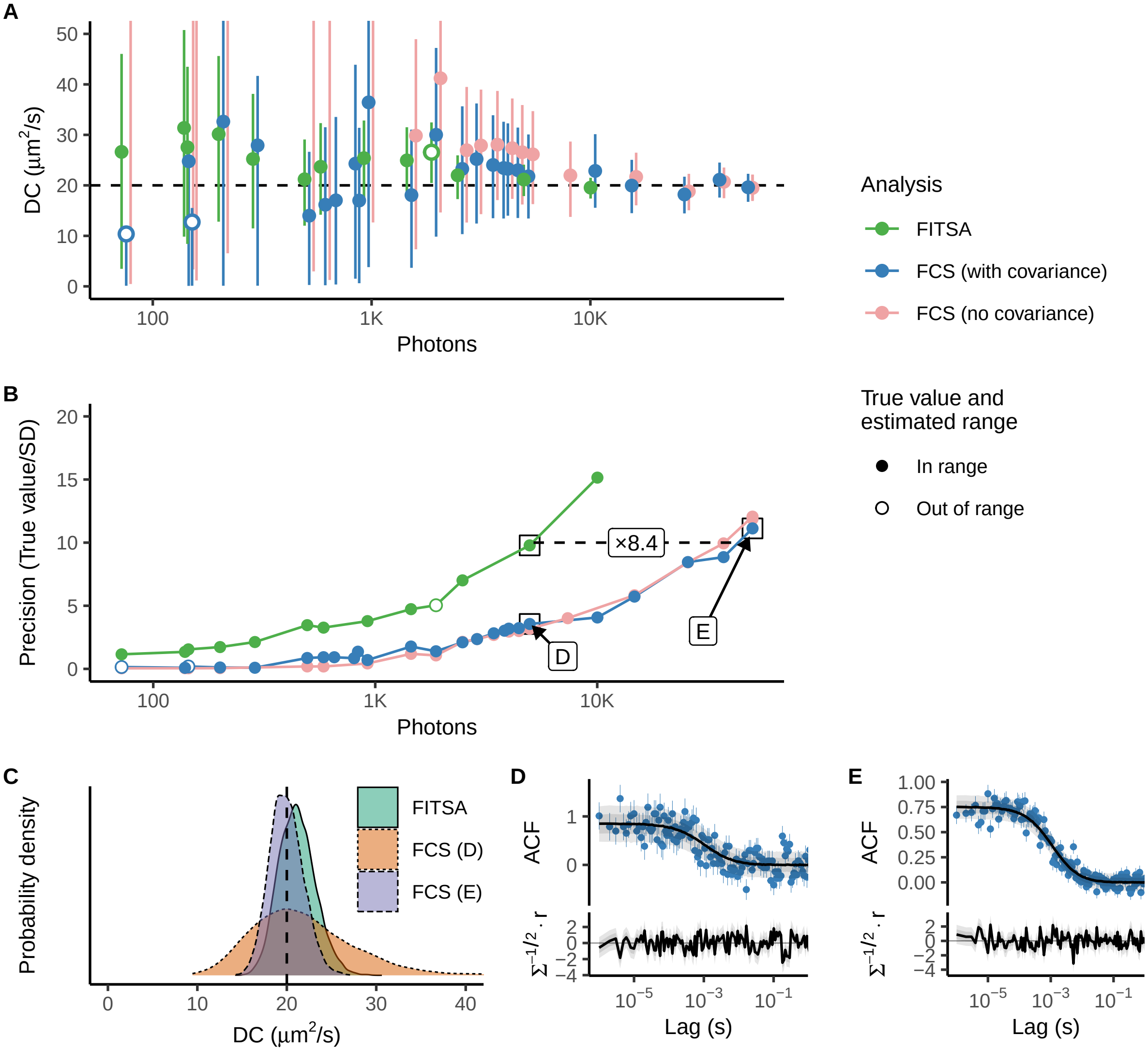

Figure S7: **Precision of estimated diffusion coefficient with the reduced molecular brightness.** Here, a synthetic trace corresponding to diffusion coefficient of 20 $\mu m^2/s$ was used with the molecular brightness of 10000 1/s. These data were used while composing a summary of molecular brightness impact shown in Fig. 5. A: The estimated DC is plotted as a function of photons in the trace exceeding background emission. B: Precision of DC estimates by different methods is shown as the true value divided by the standard deviation (SD). Estimates marked by black boxes were selected to illustrate the posterior probability density for DC estimated by FITSA and FCS. C: Posterior probability densities for DC estimates marked in B. D, E: ACF and its predictive posterior by FCS model are shown for selected FCS estimates. Below the fit, standardized residuals are shown (r – residual; Σ – covariance).

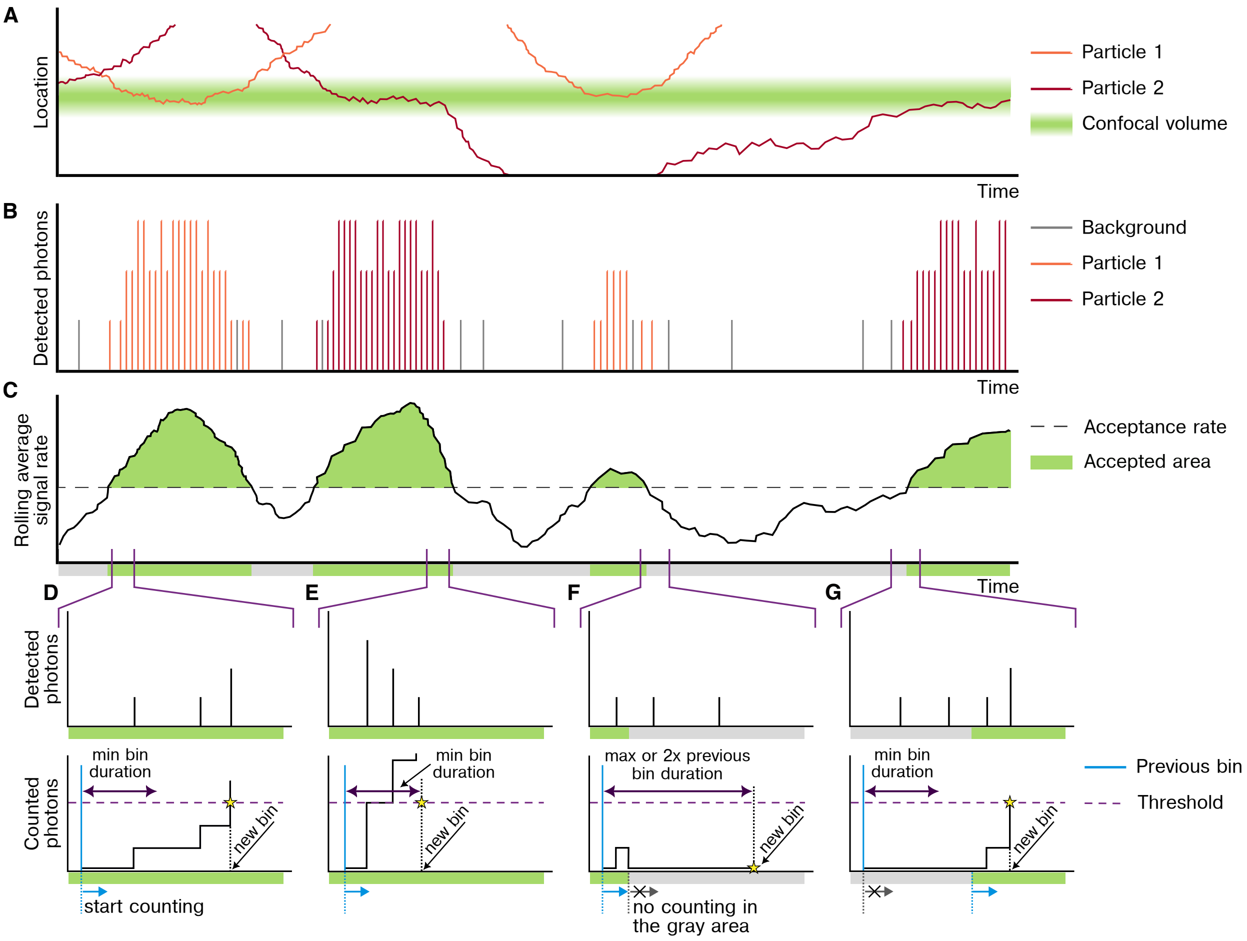

Figure S8: **Signal binning strategy in FITSA.** A and B: Movement of the particles next to the laser-illuminated confocal volume (A) and photon detection trace (B). C: Signal rolling average analysis showing accepted areas (green) where photon counts exceed the background threshold and determine bin sizes. D-G: Representative scenarios encountered during signal binning, with detected photons (top) and accumulated counts (bottom): D: Standard binning within accepted area using photon threshold, E: High emission rate requiring minimum bin duration, F: Exit from accepted area with progressive bin size increase, G: Entry into accepted area with new photon count accumulation. Detailed explanation provided in the main text.

Table S1: **Probability distributions.**

Distribution	Notation	Probability density function	Mean	Variance
Poisson	$Poisson(\lambda)$	$\frac{\lambda^x e^{-\lambda}}{x!}$	λ	λ
Normal	$Normal(\mu, \sigma^2)$	$\frac{1}{\sqrt{2\pi\sigma^2}} e^{-\frac{(x-\mu)^2}{2\sigma^2}}$	μ	σ^2
Gamma	$Gamma(\alpha, \beta)$	$\frac{\beta^\alpha}{\Gamma(\alpha)} x^{\alpha-1} e^{-\beta x}$	$\frac{\alpha}{\beta}$	$\frac{\alpha}{\beta^2}$
Inverse Gamma	$InvGamma(\alpha, \beta)$	$\frac{\beta^\alpha}{\Gamma(\alpha)} x^{-\alpha-1} e^{-\frac{\beta}{x}}$	$\frac{\beta}{\alpha-1}$	$\frac{\beta^2}{(\alpha-1)^2(\alpha-2)}$

Table S2: **Parameter values used for the generation of the synthetic traces.**

**Categories**	**Particles**		**Timing**		**Emission Rates**		**Box**		**PSF**	
**Parameters**	D	K	T_{tot}	Δt	μ_{bg}	μ_{mol}	B_{xy}	B_z	ω_{xy}	ω_z
**Units**	$\mu m^2 s^{-1}$	-	s	s	s^{-1}	s^{-1}	μm	μm	μm	μm
Fig 2, 3	10	25	1	10^{-6}	10^3	10^5	4	6	0.3	1.1
Fig 4, S1, S2	20	25	100	10^{-6}	10^3	10^5	4	6	0.3	1.1
Fig S3	200	200	100	10^{-6}	10^3	10^5	20	30	0.3	1.1
Fig S4	2	25	100	10^{-6}	10^3	10^5	4	6	0.3	1.1
Fig 5, S5	20	25	100	10^{-6}	10^3	$5 \cdot 10^4$	4	6	0.3	1.1
Fig 5, S6	20	25	100	10^{-6}	10^3	$2.5 \cdot 10^4$	4	6	0.3	1.1
Fig 5, S7	20	25	100	10^{-6}	10^3	$1 \cdot 10^4$	4	6	0.3	1.1

Table S3: **Parameter values used for the analyses of the synthetic traces and the experimental data.**

Categories	Priors					PSF			Signal Binning			Signal Splitting		
**Parameters**	N	μ_{bg}	μ_{mol}	B_{xy}	B_z	ω_x	ω_y	ω_z	I_{thr}	Δt_{min}	Δt_{max}	N_S^{min}	N_S^{max}	Δt_S
**Units**	-	s^{-1}	s^{-1}	μm	μm	μm	μm	μm	-	s	s	-	-	s
Fig 2, 3	1	1000	10^5	4	8	0.3	0.3	1.1	1	10^{-4}	10^{-2}	50	1000	10^{-3}
Fig 4, S2	1	1000	10^5	4	8	0.3	0.3	1.1	1	10^{-5}	10^{-2}	50	1000	$5 \cdot 10^{-4}$
Fig S1	1	1000	$2 \cdot 10^4$	4	8	0.3	0.3	1.1	1	10^{-5}	10^{-2}	50	1000	$5 \cdot 10^{-4}$
Fig S3	1	1000	10^5	4	8	0.3	0.3	1.1	1	10^{-5}	10^{-2}	50	1000	$5 \cdot 10^{-4}$
Fig S4	1	1000	10^5	4	8	0.3	0.3	1.1	1	10^{-4}	10^{-2}	50	1000	10^{-3}
Fig 5 50K 25K	1	1000	10^5	4	8	0.3	0.3	1.1	1	10^{-5}	10^{-2}	50	1000	10^{-3}
Fig 5 10K	1	1000	10^5	4	8	0.3	0.3	1.1	1	10^{-5}	10^{-2}	150	1000	10^{-3}
Fig 6	1	1000	10^5	4	8	0.3	0.3	1.1	1	10^{-4}	10^{-2}	50	1000	10^{-3}
Fig 7 ($1nM$ / $19\mu W$)	1	5000	10^5	4	8	0.3	0.4	0.9	1	10^{-5}	10^{-2}	50	200	10^{-3}
Fig 7 ($1nM$ / $11\mu W$)	1	3500	10^5	4	8	0.3	0.4	0.9	1	10^{-5}	10^{-2}	50	200	10^{-3}
Fig 7 ($1nM$ / $4\mu W$)	1	1500	10^5	4	8	0.3	0.4	0.9	1	10^{-5}	10^{-2}	50	200	10^{-3}
Fig 7 ($0.1nM$ / $19\mu W$)	1	1000	10^5	4	8	0.3	0.4	0.9	1	10^{-5}	10^{-2}	50	1000	10^{-3}
Fig 7 ($0.1nM$ / $11\mu W$)	1	800	10^5	4	8	0.3	0.4	0.9	1	10^{-5}	10^{-2}	50	1000	10^{-3}
Fig 7 ($0.1nM$ / $4\mu W$)	1	350	10^5	4	8	0.3	0.4	0.9	1	10^{-5}	10^{-2}	50	1000	10^{-3}
Fig 8	1	3000	10^5	4	8	0.3	0.32	0.93	1	10^{-5}	10^{-2}	50	1000	10^{-3}
